# Anti-CD37 radioimmunotherapy with ^177^Lu-NNV003 synergizes with the PARP inhibitor olaparib in treatment of non-Hodgkin’s lymphoma *in vitro*

**DOI:** 10.1371/journal.pone.0267543

**Published:** 2022-04-29

**Authors:** Marion M. Malenge, Astri Fjelde Maaland, Ada Repetto-Llamazares, Brian Middleton, Marcel Nijland, Lydia Visser, Sebastian Patzke, Helen Heyerdahl, Arne Kolstad, Trond Stokke, Anne Hansen Ree, Jostein Dahle

**Affiliations:** 1 Nordic Nanovector ASA, Oslo, Norway; 2 Institute of Clinical Medicine, University of Oslo, Oslo, Norway; 3 Department of Radiation Biology, Institute for Cancer Research, Norwegian Radium Hospital, Oslo University Hospital, Oslo, Norway; 4 Inferstats Consulting Ltd, Cheshire, United Kingdom; 5 University Medical Center Groningen, Groningen, The Netherlands; 6 Department of Oncology, Innlandet Sykehus, Lillehammer, Norway; 7 Department of Oncology, Akershus University Hospital, Lørenskog, Norway; Columbia University, UNITED STATES

## Abstract

**Background and purpose:**

PARP inhibitors have been shown to increase the efficacy of radiotherapy in preclinical models. Radioimmunotherapy results in selective radiation cytotoxicity of targeted tumour cells. Here we investigate the combined effect of anti-CD37 β-emitting ^177^Lu-NNV003 radioimmunotherapy and the PARP inhibitor olaparib, and gene expression profiles in CD37 positive non-Hodgkin’s lymphoma cell lines.

**Materials and methods:**

The combined effect of ^177^Lu-NNV003 and olaparib was studied in seven cell lines using a fixed-ratio ray design, and combination index was calculated for each combination concentration. mRNA was extracted before and after treatment with the drug combination. After RNA-sequencing, hierarchical clustering was performed on basal gene expression profiles and on differentially expressed genes after combination treatment from baseline. Functional gene annotation analysis of significant differentially expressed genes after combination treatment was performed to identify enriched biological processes.

**Results:**

The combination of olaparib and ^177^Lu-NNV003 was synergistic in four of seven cell lines, antagonistic in one and both synergistic and antagonistic (conditionally synergistic) in two, depending on the concentration ratio between olaparib and ^177^Lu-NNV003. Cells treated with the combination significantly overexpressed genes in the TP53 signalling pathway. However, cluster analysis did not identify gene clusters that correlate with the sensitivity of cells to single agent or combination treatment.

**Conclusion:**

The cytotoxic effect of the combination of the PARP inhibitor olaparib and the β-emitting radioimmunoconjugate ^177^Lu-NNV003 was synergistic in the majority of tested lymphoma cell lines.

## Introduction

Non-Hodgkin’s Lymphoma (NHL) is the most common haematological malignancy and is classified into different histologic subtypes [1]. Among the aggressive NHLs diffuse large B-cell lymphoma (DLBCL) can be subdivided into activated B-cell (ABC) and germinal centre (GCB) DLBCL based on either immunostaining or gene expression profiling [1]. Mantle cell lymphoma (MCL) is a distinct and more uncommon NHL [1]. NHL occurs as a consequence of genetic alterations occurring during the error prone process of B-cell differentiation and maturation. The resulting lymphomas often have deficiencies in DNA damage response (DDR) pathways linked to mutations in *ATM*, *PTEN* and *TP53* tumour suppressor genes [2–4]. Malignant cells utilise compensatory DNA repair strategies to prevent catastrophic DNA damage. Targeting these complementary DNA repair pathways results in dysfunction of both DNA repair pathways, inducing synthetic lethality [5, 6].

Olaparib inhibits the DNA repair enzymes poly (ADP ribose) polymerase 1 and 2 (PARP1 and PARP2), which are activated in response to DNA single strand breaks (SSB) [7]. Consequently, the PARPs are unable to recruit DNA repair proteins and are trapped at the SSB site causing stalling and collapse of the DNA replication fork which results in cytotoxic double strand breaks (DSB) [8]. Olaparib has been approved by the FDA for *BRCA* mutated ovarian and breast cancer. The *BRCA* mutation causes impairment of DNA DSB repair, making the cells harbouring this mutation sensitive to olaparib. Olaparib has also been shown to be effective in preclinical models of MCL harbouring *ATM* mutation [9], which is present in 41–56% of MCL and 13–20% of DLBCL patients [10–12], and also impairs the DSB repair pathway. The PARP inhibitor veliparib has shown clinical activity in NHL in combination with the alkylating agent bendamustine and the anti-CD20 antibody rituximab [13].

Radiation induces cytotoxic DNA lesions in form of SSB or DSB, where the latter is more lethal. Combination of radiation with PARP inhibition results in the transformation of the induced SSBs to DSBs, increasing the cytotoxic effect of the treatments. Several preclinical studies have shown that PARP inhibitors sensitise tumour cells to radiation [14–24] and combine synergistically with antibody-drug conjugates [25]. The combination of the anti-EGFR antibody cetuximab, olaparib and radiation has been studied in patients with head and neck squamous cell carcinoma [26] and there are currently several phase 1 studies ongoing investigating olaparib in combination with radiotherapy in patients with glioblastoma, lung cancer, breast cancer and head and neck squamous cell carcinoma [27–29].

Radioimmunotherapy (RIT) delivers targeted radiation that induces DNA damage, priming malignant cells for apoptosis with limited toxicity to normal tissue. We have developed a next generation RIT, ^177^Lu-NNV003, for treatment of B-cell malignancies. It consists of a chimeric mouse-human anti-CD37 antibody (NNV003), conjugated with p-SCN-Bn-DOTA (DOTA) that chelates the ß-emitting radionuclide lutetium-177 [30]. The murine version of ^177^Lu-NNV003; ^177^Lu-lilotomab satetraxetan, is currently in clinical testing for treatment of relapsed/refractory follicular lymphoma (NCT01796171) and DLBCL (NCT02658968).

In the present study, we aimed to determine the *in vitro* cytotoxicity and phenotypic outcomes of combining ^177^Lu-NNV003 with olaparib in DLBCL and MCL cell lines.

## Materials and methods

### Labelling and quality control of antibodies with ^177^Lu

NNV003 (IgG_1_, mouse variable regions, κ, and human constant region, κ) was conjugated with p-SCN-Bn-DOTA (Macrocyclics, USA) and labelled with ^177^Lu as previously described [30]. Briefly, the pH of DOTA-NNV003 was adjusted to 5.4 using 0.25 M ammonium acetate buffer and ^177^Lu in 10 mM HCl (ITG, Germany) was added to obtain specific activity of approximately 550 MBq/mg. The sample was incubated for 30 min at 37˚C and then diluted in a solution of 0.3% Tween 20 (VWR, USA) and 20% Glycerol (Merck KGaA, Germany). Radiochemical purity above 95% was verified by instant thin layer chromatography (Tec-Control ITLC strips, Biodex Medical, USA) and the immunoreactivity was verified using a modified Lindmo model [31] using a standardised setup with one cell concentration of 75 x10^6^ Ramos cells/ml.

### Cell lines

The MCL cell lines REC-1 and GRANTA-519, the GCB-DLBCL cell lines DOHH-2, SU-DHL-4, and WSU-DLCL-2, the ABC-DLBCL cell lines U-2932 and OCI-LY-10 and the Acute Lymphocytic Leukemia (ALL) cell line REH were used in this study. REC-1, DOHH-2, SU-DHL-4, WSU-DLCL-2, REH and U-2932 were cultured in RPMI medium, GRANTA-519 was cultured in DMEM medium and OCI-LY-10 was cultured in IMDM medium. The media were supplemented with 15% (OCI-LY-10) or 10% (all others) heat inactivated foetal bovine serum and 1% penicillin/streptomycin (media and supplement from Thermo Fisher Scientific, USA). All cell lines were provided by University Medical Center Groningen (Netherlands), except OCI-LY-10, which was kindly provided by Institute of Oncology Research (Switzerland) and REH which was acquired from ATCC (LGC Standards, Wesel, Germany). Cell lines were kept in exponential growth by cell subculturing every 3–4 days and all in vitro studies were started on day 3 after cell subculturing.

### CD37 expression

CD37 expression of all cell lines was investigated using fluorescently labeled anti-CD37 antibody NNV003 and Flow Cytometry (FC). The aim of the study was to explore if CD37 expression co-varied with sensitivity to ^177^Lu-NNV003 or the combination outcomes of the different cell lines used.

NNV003 was labeled with Alexa Fluor 647 (AF-647) using the Alexa Fluor 647 Protein Labeling Kit (Thermo Fisher, A20173) following the manufacturer’s protocol.

1 ml of cell suspension was transferred to 5 ml FC tubes, washed once and resuspended in 50 μl of PBS + 0,5% Bovine Serum Albumin (BSA) (VWR Chemicals, 421501J). Cells were then incubated with 10 μg/ml NNV003-AF647 during 30 min at 4°C. In order to assess non-specific binding cells pre-incubated with 1 mg/ml NNV003 for 30 min at 4°C were used. Autofluorescence was evaluated by measuring untreated cells (blanks). After incubation with NNV003-AF647 cells were washed once more to remove unbound antibody and resuspended in 0.7–0.8 ml of PBS + 0,5% BSA.

Samples were acquired using Guava easyCyte 12HT (Merck Millipore) using a 642nm wavelength laser and the fluorescence emitted was detected at 661nm using a 15nm bandpass filter. The gain of the detector was set so that the peak intensity from the blank samples was placed in the first decade of the logarithmic scale. In addition, SS and FS signals were detected.

The collected data was analyzed using GuavaSoft InCyte (Merck Millipore). Gating on the FS-SS dot-plot was used to select the cell population of interest and further gating on the SS Width against SS Area dot plot was used to select single cells. Mean Fluorescence Intensity (MFI) of all samples was obtained, and further calculations were done in Microsoft Excel.

The REH cell line was used as negative control. MFI from all cell lines was divided by the MFI of the negative control to assess Relative MFI. The experiment was repeated a total of 2 times and the results are presented as Average ± Standard Deviation of both experiments. One way ANOVA with multiple comparisons (Fisher LSD test) with a threshold of p<0.05 was used to compare the relative MFI of the cell lines.

### Sensitivity to single agents

Olaparib (Selleck Chemicals USA) was dissolved in DMSO, aliquoted and stored at -20˚C.

Cells were seeded in 96-deep-well plates at concentrations of 2 mill/ml for OCI-LY-10, GRANTA-519 and U-2932 and 8 mill/ml for REC-1, DOHH-2, SU-DHL-4 and WSU-DLCL-2. Using a digital drug dispenser (D300e, TECAN, Switzerland), 1.3 nM– 316 μM of olaparib or 0.09 ng/ml– 88.5 μg/ml (50 Bq/ml– 50 MBq/ml) of ^177^Lu-NNV003 was randomly added to the wells (total cell suspension volume of 100 μl) in triplicates. The cells were incubated for 20–24 h while shaking at 37°C and 5% CO_2_. The cells were diluted 200x in cell culture medium to decrease the amount of ^177^Lu-NNV003 in the medium and the wells containing olaparib were refilled to maintain the initial drug concentration. 50 μl of the diluted cell suspension was transferred to 384-well-plates for further growth for 3 days, after which they were added 50 μl of RealTime-Glo™ MT Cell Viability Assay (Promega, USA). Luminescence, proportional to the number of viable cells, was measured after 1 hour, 24 hours and 48 hours (days 3, 4 and 5 after treatment initiation) using a Spark microplate reader (TECAN, Switzerland). Relative viability was calculated by dividing the luminescence values from treated cells by luminescence from non-treated cells. The relative viability was plotted against drug concentration and sigmoidal curve fitting (four-parameter logistic curves) was performed in GraphPad Prism 8.00 (GraphPad Software, USA). IC50, area under the curve (AUC) and point viabilities [32] were used to estimate sensitivity to the drugs. The point viabilities for olaparib were measured at 21.6 μM, corresponding to the maximum achievable clinical plasma concentration at recommended monotherapy dose [33]. The point viabilities for ^177^Lu-NNV003 were measured at 250 ng/ml, which is close to the average IC50 for the drug across the cell lines. Results are presented as Mean ± Standard Deviation from 2 to 5 independent experiments.

### Combination study

A fixed-ratio ray design [34] was used to study the effect of combining ^177^Lu-NNV003 with olaparib. Briefly, the two drugs were mixed together at a constant ratio (Z) following Eq 1. Each combination Z is defined by a fraction, f, between 0 and 1, where f equal to 0 or 1 corresponds to only olaparib or ^177^Lu-NNV003, respectively. Three combinations were made using f = 0.25, 0.5 and 0.75. To obtain a dose response curve of the combinations, 9 concentrations of Z were used by multiplying Eq 1 with factors ranging between 0.003 and 150 depending on cell line. See S1 Table for concentrations used in the experiment. The experimental procedure was the same as previously described for evaluation of sensitivity to single agents.


$$Z=f\;\times {\;}^{177}LuNNV003{\;}_{IC50}+\left(1-f\right)\times olaparib_{IC50}$$ (1)

The order and timing of the administration of the treatments was based in a pilot study using GRANTA-519 cells where it was tested whether adding olaparib four hours before, 24 hours after or simultaneously with ^177^Lu-NNV003 had any effect on the combination outcome. The experimental procedure was the same as previously described.

### Analysis of ray design

The relative cell survival was calculated by dividing the luminescence values of treated cells by the luminescence values of non-treated cells. 1 minus this ratio was taken to represent the proportion of killed cells. The bottom asymptote of the dose response curve was fixed to 0 and the top asymptote was set to be less than or equal to 1. Sigmoid curves (3-parameter logistic curves) were fitted for each ray, with the assumption that the variability about the fitted curve would be similar for all rays, allowing the use of a global model [34]. The variance was dependent on the response, so to account for this effect the variance was modelled for each dose using Eq 2

$$var=c^{2}\;\times response^{p}$$ (2)

where the response is the proportion of cells killed at that dose, and c and p are parameters from the global model. The curve fitting was done using SAS/STAT 14.1 software in SAS Version 9.4, in particular PROC NLMIXED. Combination indices (CInd) were calculated per concentration using a model based on different maximum effects of the drugs, and unequal Hill slopes of the dose response curves [35], derived by Grabovsky and Tallarida [36]. CInd for concentrations leading to 0% cell death were regarded as not relevant and excluded from the analysis. A point was considered significantly synergistic or antagonistic if the 95% confidence interval of the CInd was below or above 1, respectively. If the CInd was below 0.85 or above 1.15 and the 95% confidence included 1, the point was considered non-significantly synergistic or antagonistic. Points were considered additive between 0.85 and 1.15, and if the 95% confidence interval was within this range, it was considered significant. Some dose response curves could not be well fitted and therefore the CInd could not be calculated and these points are presented as missing data. Results are presented for each independent experiment (N = 1–2) or as average ± SD of all statistically significant CInds for each day.

### Gene expression analysis

The cells were treated for 24 hours with the combination of the drugs at concentrations corresponding to their monotherapy (see S1 Table for treatment concentrations). The cells including untreated controls were washed and total RNA isolated using RNeasy mini kit (Qiagen^©^, Germany) following the manufacturers’ protocol.

RNA integrity was verified using 2100 Bioanalyzer (Agilent Technologies Inc., CA, USA) and adjusted to an acceptable concentration. Libraries were generated from the RNA using Illumina stranded mRNA kit (Illumina Inc., CA, USA) and sequenced on an Illumina NextSeq500 system (Illumina Inc., CA, USA) using 75 bp single reads. Obtained reads were aligned against the reference human genome (UCSC hg19) using STARalign v2.5.0.

Genes that were expressed at very low levels were excluded from the analysis by a cut-off of 10 normalised reads, applied to the sum of gene expression at baseline and in the respective treated samples. This reduced the gene list data from 23,269 genes to 6,054 genes (S4 Fig).

The resulting 6,054 genes were log2-transformed and genes with a standard deviation larger than 1 of the baseline expressions in the 7 cell lines were included for further analysis, which resulted in 559 genes that were then min-max normalised to scale the entire dataset to a 0 to 1 range.

Hierarchical cluster analysis on the baseline genes was performed on the scaled data using Morpheus software (broadinstitute.org). For this analysis, Euclidean distance and complete linkage were used to compute the distance between clusters. This analysis was used to visualise the correlation of clusters to cell line histology subtypes, drug sensitivity and drug combination outcome.

Differential gene expression between the untreated cell lines and the corresponding combination treated samples was analysed using Cufflinks and Cuffdiff v2.2.1. To identify genes that were significantly up- or down- regulated after treatment with the combination of ^177^Lu-NNV003 and olaparib, a threshold was set on the log2 fold change (FC) >0.5 or <-0.5 and p <0.05 compared to baseline (S4 Fig).

Functional and pathway enrichment analysis was done using the web-based functional annotation tool: DAVID 6.8 (david.ncifcrf.gov) [37]. The *Homo sapiens* genome was selected as the background and the differentially expressed genes mapped against it. Gene Ontology (GO) biological process terms and Kyoto Encyclopedia of Genes and Genomes (KEGG) pathway with p <0.05, count ≥2 and FDR < 1% were considered to be statistically significant. The GO terms were matched against the outcome of drug combinations to identify their correlation and contribution towards the assigned synergy score.

### Gene mutation analysis

To identify mutations in expressed genes related to DNA damage repair, mutation calling was done using Isaac Variant Caller version 2.3.13–31 c98c29-dirty and hg19 reference. Variant calls passing all quality requirements were annotated using VEP Ensembl GRCh37 release 98.

## Results

### CD37 expression

**Fig 1:**
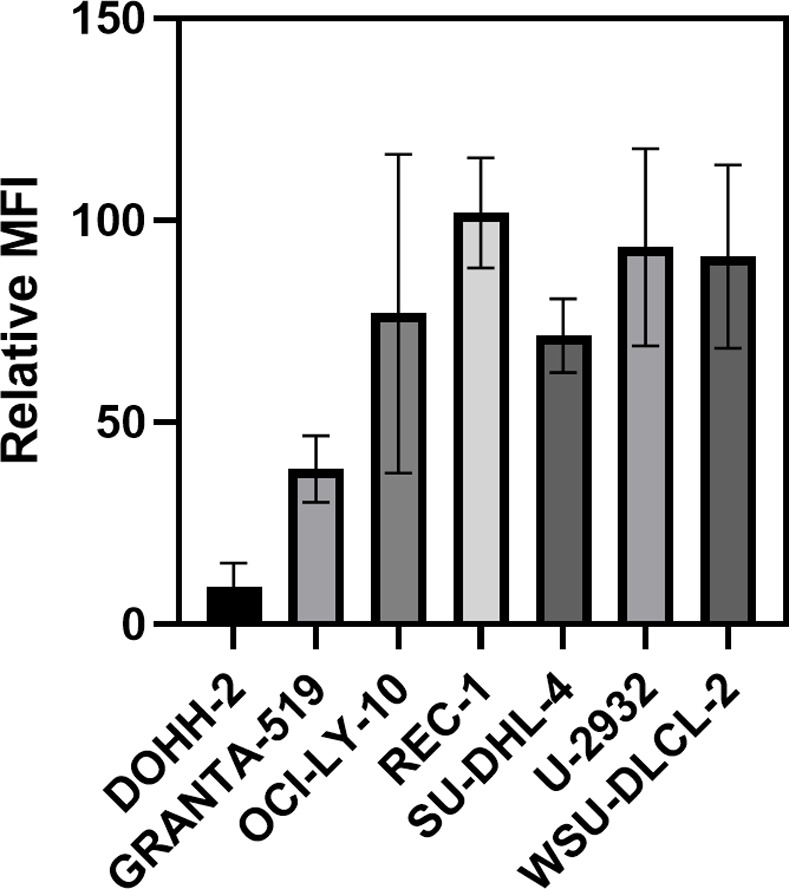
CD37 expression in seven cell lines measured by flow cytometry. Mean Fluorescence Intensitiy (MFI) of Mantle Cell Lymphoma cell lines (GRANTA-519 and REC-1) and DLBCL cell lines (DOHH-2, OCI-LY-10, SU-DHL-4, U-2932 and WSU-DLCL-2) relative to MFI of the Acute Lymphocytic Leukemia cell line REH used as negative control. Results were obtained from Flowcytometry measurements using NNV003 labeled to AlexaFlour 647 to measure CD37 expression. Cell suspensions were incubated with 10 μg/ml NNV003-AF647 for 30 min at 4°C. Cells were analysed on a Guava easyCyte 12HT flowcytometer (Merck Millipore) using GuavaSoft InCyte (Merck Millipore) for data acquisition. Data is presented as Mean ± SD, N = 2.

Binding of NNV03 to the seven different cell lines was measured so as to assess CD37 surface expression. DOHH2 and GRANTA-519 showed the lowest CD37 expression (p<0.05 for DOHH2 vs. all cell lines except GRANTA-519; p<0.05 for GRANTA-519 vs. Rec-1, U2932 and WSU-DLCL2, Fig 1). The remaining five cell lines (SUDHL-4, OCI-LY-10, WSU-DLCL2, U2932 and REC-1) showed between 70 to 100 times higher binding intensity than the negative control REH (a pre-B-cell Acute Lymphoblastic Leukemia cell line) but differences were not statistically significant. S1 and S2 Figs show representative gating and histograms used for the CD37 expression analysis respectively.

### Single-agent sensitivity

**Fig 2:**
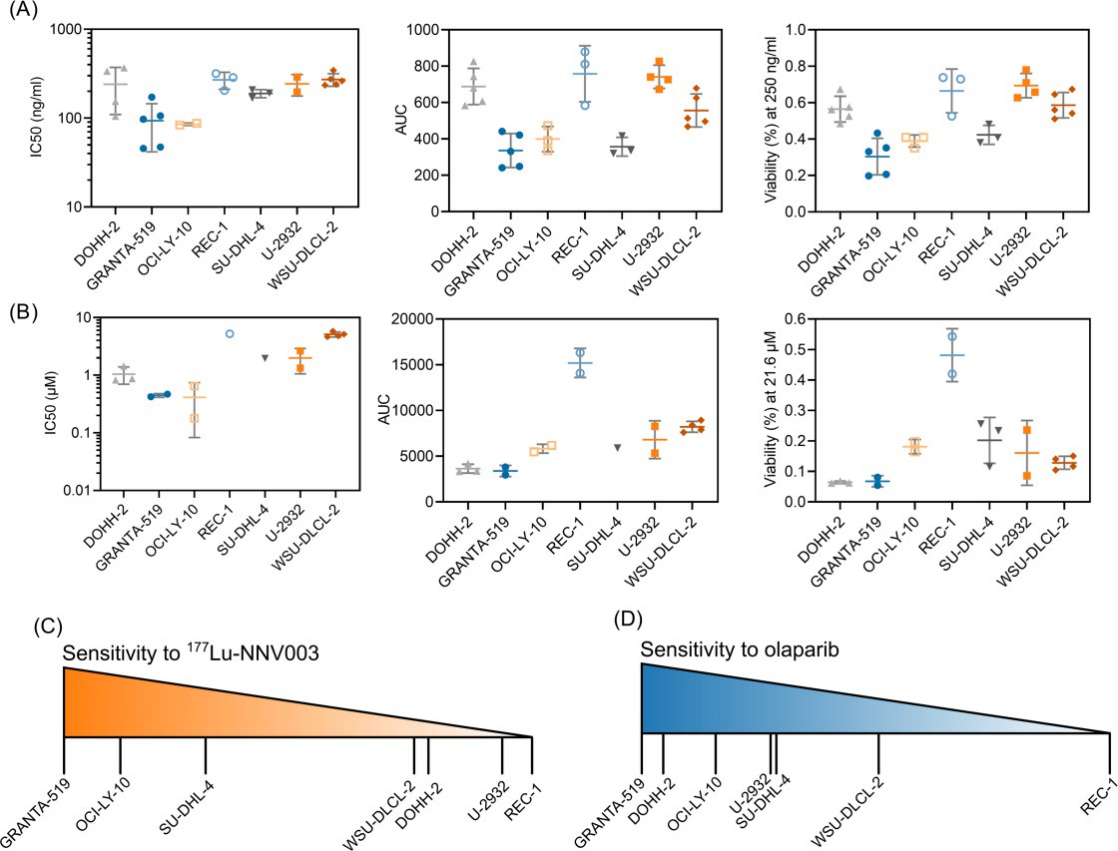
Single agent sensitivity of lymphoma cell lines to ^177^Lu-NNV003 and olaparib. Sensitivity of Mantle Cell Lymphoma cell lines (GRANTA-519 and REC-1) and DLBCL cell lines (DOHH-2, OCI-LY-10, SU-DHL-4, U-2932 and WSU-DLCL-2) to (A) ^177^Lu-NNV003 and (B) olaparib as single agents, as measured at day 5. The plots in (A) and (B) are derived from their respective dose-response curves and show: half maximal inhibitory concentration (IC50) (left panel), Area under the dose-response curve (AUC) (middle panel) and the individual cell viabilities (point viabilities, measured at 21.6 μM olaparib or 250 ng/ml ^177^Lu-NNV003) (right panel) as different parameters for sensitivity assessment. Data points represent results from independent experiments (N = 2–5), lines show the mean and error bars the SD. The data from (A) and (B) were normalised from 1 (most sensitive) to 0 (least sensitive), averaged and plotted on a scale as shown in (C) and (D).

To determine the sensitivity of the cell lines to ^177^Lu-NNV003 and olaparib IC50s, AUCs and individual viabilities were calculated (Fig 2 and S1 Table). The three viability estimates were in accordance and showed that the cell lines had diverse response to the single-agent treatments. To better see a trend in the responses of cell lines, the data from the three measurements were normalised from most sensitive to least sensitive and plotted on a scale. The MCL cell line GRANTA-519 was the most sensitive cell line for both drugs, whereas the other MCL cell line REC-1 (Fig 2C and 2D) was the least sensitive. For treatment with olaparib, only REC-1 was determined as resistant with around 50% viability at the maximum achievable plasma concentration of the drug. All the cell lines were classified as sensitive to ^177^Lu-NNV003 because the IC50s were below 8 μg/ml which has been reported in a previous study to be the concentration conferring resistance to ^177^Lu-lilotomab, the murine version of ^177^Lu-NNV003 [38].

### Combination of ^177^Lu-NNV003 and olaparib

In the pilot study performed with GRANTA-519 cells, negligible differences between the different schedules of administration were observed. A slight tendency towards better effect by adding olaparib at the same time or prior to ^177^Lu-NNV003 was observed (S3 Fig) and therefore simultaneous administration was chosen for all combination studies.

**Fig 3:**
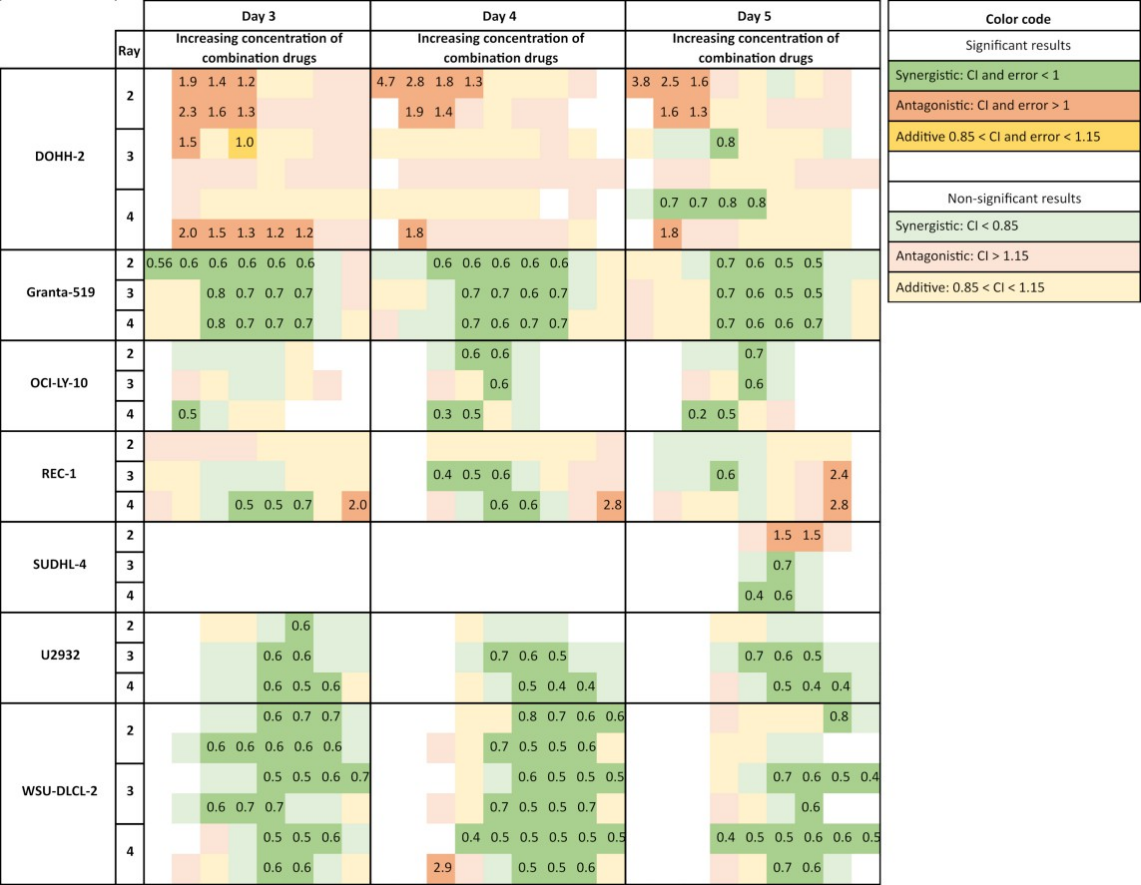
Heat map of Combination Indexes (CInd) calculated using Ray Design Analysis. Heat map showing Combination Indexes (CInd) calculated using the Ray Design Analysis based on the outcomes of the combination treatment with olaparib and ^177^Lu-NNV003 in Mantle Cell Lymphoma cell lines (GRANTA-519 and REC-1) and DLBCL cell lines (DOHH-2, OCI-LY-10, SU-DHL-4, U-2932 and WSU-DLCL-2) for all used rays. Results are presented for days 3, 4 and 5 after treatment initiation. A data point was considered significantly synergistic or antagonistic if the 95% confidence interval of the CInd was below or above 1, respectively. If the CInd was below 0.85 or above 1.15 and the 95% confidence included 1, the point was considered non-significantly synergistic or antagonistic. Points were considered additive between 0.85 and 1.15, and if the 95% confidence interval was within this range, it was considered significant. White square = data missing or non-relevant. The numbers in the squares indicate the calculated CInds where statistical significance was observed. Results presented for each independent experiment N = 1–2.

**Fig 4:**
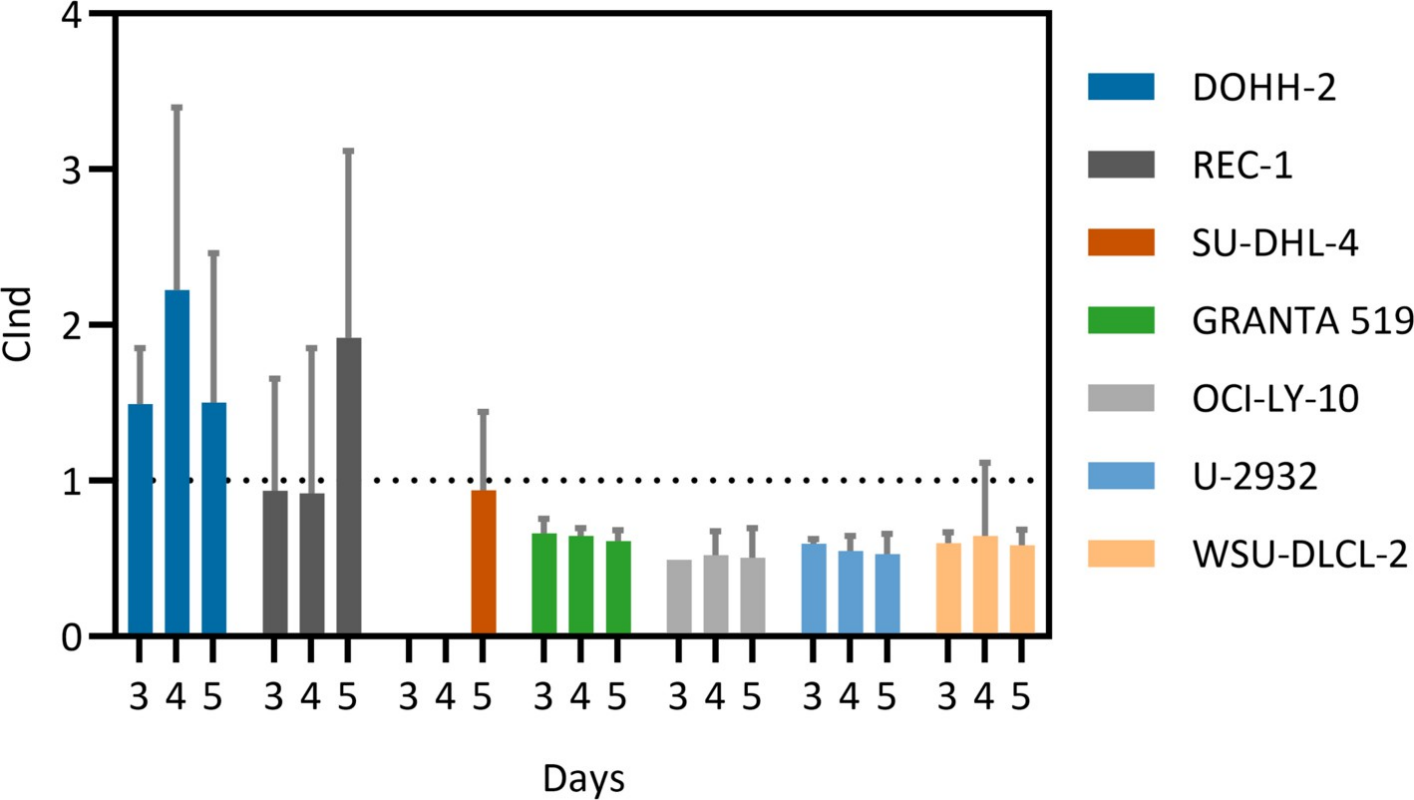
Average Combination Indexes (CInd) for all cell lines measured 3, 4 and 5 days after treatment initiation. Average ± SD of statistically significant Combination Indexes calculated using the Ray Design Analysis based on the outcomes of the combination treatments with olaparib and ^177^Lu-NNV003 in Mantle Cell Lymphoma cell lines (GRANTA-519 and REC-1) and DLBCL cell lines (DOHH-2, OCI-LY-10, SU-DHL-4, U-2932 and WSU-DLCL-2). Results are presented for days 3, 4 and 5 after treatment initiation. CInds > 1 indicates synergism, CInds < 1 indicates antagonism and CInd = 1 indicates an additive combination outcome.

To estimate the effect of the combined treatment of ^177^Lu-NNV003 and olaparib, CInd (Combination Index) were calculated for three different ratios of the drugs (f = 0.25, 0.5, 0.75, Eq 1). The dose response curves of the rays in each cell line are presented in S5 and S6 Figs. The CInd for the combination of olaparib with ^177^Lu-NNV003 varied across cell lines, rays, days of measurement and concentrations of the combination. The trend for each cell line is summarised in Fig 3. The combined effect of olaparib and ^177^Lu-NNV003 was synergistic in four out of the seven tested cell lines: GRANTA-519, OCI-LY-10, U-2932 and WSU-DLCL-2. In REC-1 and SU-DHL-4 the combination was both synergistic, at lower concentrations (REC-1) or for two of the rays (SU-DHL-4), and antagonistic, at higher concentrations (REC-1) or for one ray (SU-DHL-4). The average CInd for each day is presented in Fig 4, to classify the cell lines to an overall combination outcome. The average CInd of the combination in REC-1 and SU-DHL-4 was close to 1 (Fig 4). The combination in these cell lines was found to be both synergistic and antagonistic and were defined as conditionally synergistic in this study. The two experiments performed with the cell line DOHH-2 gave varying results in CInd. The asymptote of the dose response curve of ^177^Lu-NNV003 alone was not well defined in these experiments (S6 Fig), giving rise to a large error in the IC50 estimates. However, there is a trend of antagonism at lower concentrations and when the relative fraction of olaparib is high. At day 5, the combination tends towards synergism and additivity at higher relative fractions of ^177^Lu-NNV003. The combination treatment was therefore classified as antagonistic in this cell line (Fig 4).

### Correlation of baseline gene expression and histology, drug sensitivity, CD37 expression and combination outcomes

**Fig 5:**
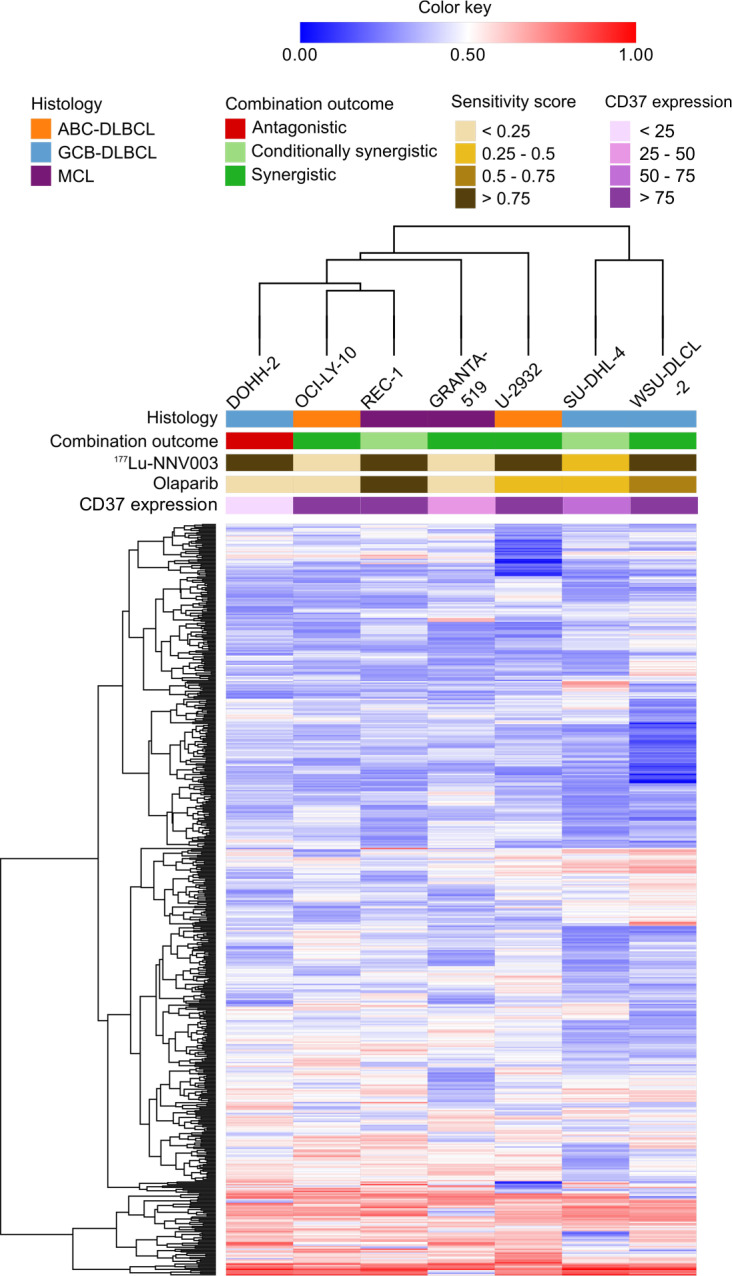
Hierarchical clustering of baseline gene expression. Unsupervised hierarchical clustering of 559 genes showing differential expression between the non-treated cell lines. The cluster groups did not reflect the NHL subtype histology of the cell lines, drug sensitivity, CD37 expression or the combination outcome. Clustering colour key indicates the intensity of normalised gene expression values.

To investigate if the baseline gene expression of the seven cell lines correlated with the outcome of the combination treatment, we performed unsupervised hierarchical cluster analysis of the 559 genes that showed differential expression between the non-treated cell lines (Fig 5). The similar heights of the different nodes indicated that none of the cell lines were more closely related to any of the others. OCI-LY-10 and REC-1 cells showed the most similar expression patterns. The cluster groups did not reflect the NHL subtype histology of the cell lines, drug sensitivity, CD37 expression or the combination outcome (Fig 5).

### Differential gene expression after combination treatment

**Fig 6:**
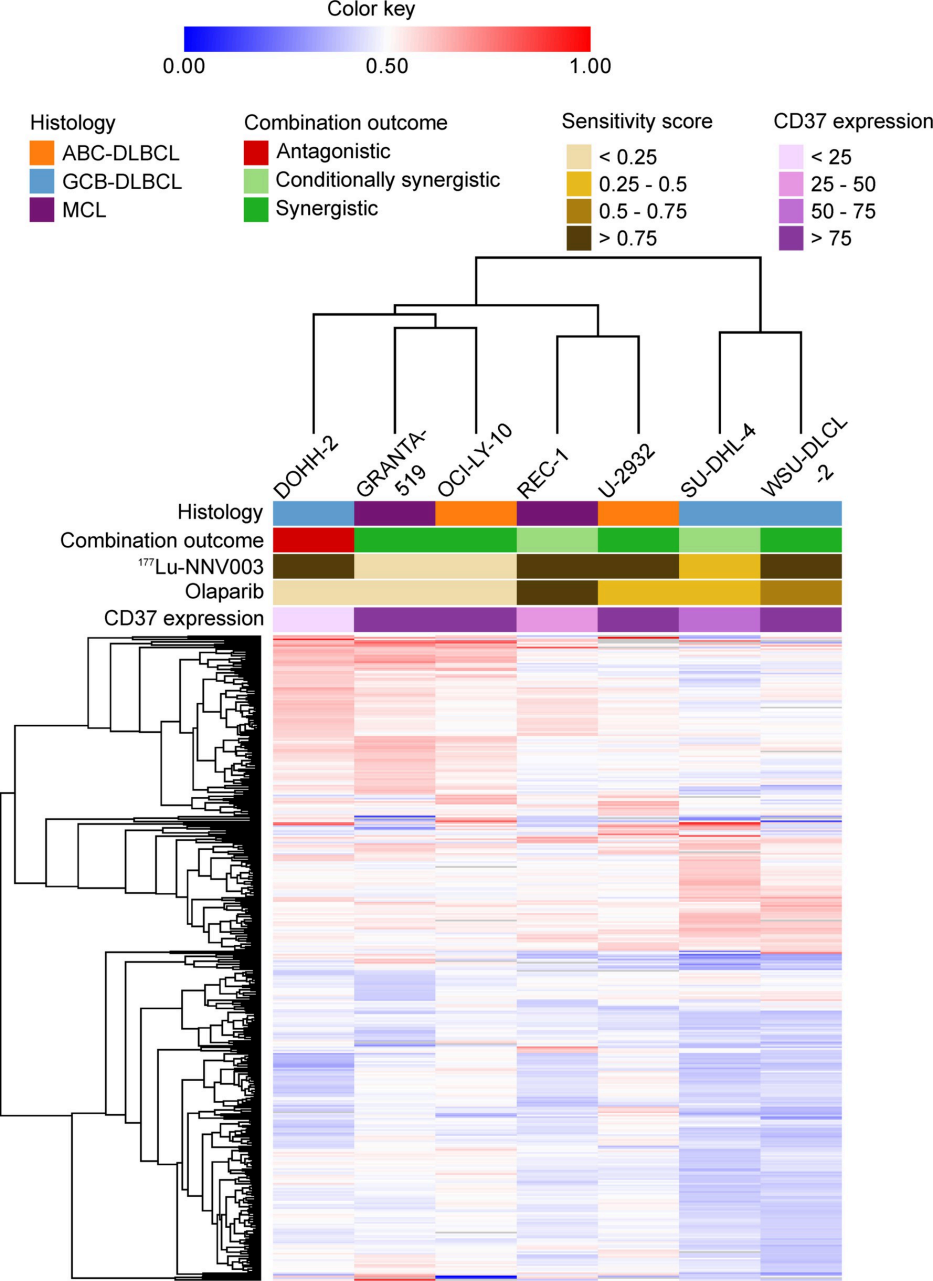
Hierarchical clustering of differentially expressed genes. Hierarchical clustering of normalised differentially expressed genes in cell lines treated with the combination of ^177^Lu-NNV003 and olaparib. There was no correlation of changes in gene expression to the combination outcome, sensitivity to single agent treatment or CD37 expression.

To identify the influence of gene expression on the outcome of the combination treatment, we compared baseline expression to gene expression after combination treatment to highlight the differentially expressed genes. The hypothesis was that these genes could provide further insight into the difference observed in the combination outcome of the different cell lines. In total, 397 genes across the cell lines were identified as differentially expressed genes 24 hours after combination treatment following the aforementioned criteria. Among them 188 genes were upregulated and 209 genes were downregulated (S2 Table). The majority of the differentially expressed genes in DOHH-2 and GRANTA-519 cells were upregulated, while the majority were downregulated in WSU-DLCL-2 and SU-DHL-4 (S2 Table). Cluster analysis of the differentially expressed genes did not identify gene clusters that correlate with the sensitivity to single agent treatment, CD37 expression or the combination outcomes (Fig 6).

### Functional and pathway enrichment of differentially expressed genes after treatment with the combination

Although the unsupervised clustering of differentially expressed genes did not identify gene clusters that correlate with the combination outcome (Fig 6), we further investigated functional annotations of the differentially expressed genes to see if they could explain the observed outcomes of the combination treatment in the different cell lines. Functional gene annotation of the upregulated and downregulated genes identified genes that were predominantly associated with enriched GO biological processes and KEGG pathways for each cell line.

Upregulated genes in DOHH-2, GRANTA-519 and OCI-LY-10 cells were commonly associated with p53 mediated DNA damage response and intrinsic apoptotic signalling, all significantly enriched in the p53 signalling pathway (Table 1). The genes: *CDKN1A*, *DDB2* and *SESN1* had the highest log2 fold change of 1.5, 1.1 and 1.1 respectively (S3 Table) in GRANTA-519 cells while *MDM2* had the highest log2 fold change of 1.0 in DOHH-2 cells. Of the three cell lines, OCI-LY-10 had the lowest fold change in these genes. *CDKN1A* encodes a cyclin-dependent kinase inhibitor which functions as a regulator of cell cycle progression, mediating the p53-dependent cell cycle G_1_ phase arrest, apoptosis and DNA repair in response to DNA damage [39].

**Table 1:** Enriched pathways in upregulated genes.

Cell line	Term	Biological process	Upregulated ‘hit’ genes	p value
DOHH-2	GO:0006977	DNA damage response, signal transduction by p53 class mediator resulting in cell cycle arrest	CDKN1A, E2F7, MDM2, ZNF385A, ATM	0.00006
	GO:0006974	Cellular response to DNA damage stimulus	CDKN1A, ZMAT3, ATM RPS27L, HERC2, ZNF385A	0.00069
	GO:0002040	Sprouting angiogenesis	NOTCH1, E2F7, RNF213	0.00323
	GO:0043065	Positive regulation of apoptotic process	ARHGEF3, NOTCH1, ATM ZMAT3, PRKDC, PHLDA3	0.00345
	GO:0042771	Intrinsic apoptotic signalling pathway in response to DNA damage by p53 class mediator	CDKN1A, RPS27L, PHLDA3	0.00494
	hsa04115	p53 signalling pathway	CDKN1A, ZMAT3, DDB2, MDM2, SESN1, ATM	0.000003
GRANTA-519	GO:0006977	DNA damage response, signal transduction by p53 class mediator resulting in cell cycle arrest	TRIAP1, CDKN1A, BTG2, E2F7, BAX, MDM2, GADD45A	0.00000
	GO:0042771	Intrinsic apoptotic signalling pathway in response to DNA damage by p53 class mediator	CDKN1A, AEN, RPS27L, PHLDA3	0.00013
	GO:0043065	Positive regulation of apoptotic process	ARHGEF3, NOTCH1, ZMAT3, BAX, ID3, GADD45A, PHLDA3	0.00039
	hsa04115	p53 signalling pathway	PPM1D, CDKN1A, BBC3, ZMAT3, BAX, DDB2, MDM2, SESN1, GADD45A	0.00000
OCI-LY-10	GO:0006977	DNA damage response, signal transduction by p53 class mediator resulting in cell cycle arrest	CDKN1A, BAX, MDM2, ZNF385A	0.000038
	GO:0006974	Cellular response to DNA damage stimulus	CDKN1A, BBC3, ZMAT3, RPS27L, ZNF385A	0.000061
	GO:0072332	Intrinsic apoptotic signalling pathway by p53 class mediator	ZMAT3, BAX, EDA2R	0.000375
	GO:0097193	Intrinsic apoptotic signalling pathway	CDKN1A, BBC3, BAX	0.000464
	hsa04115	p53 signalling pathway	CDKN1A, BBC3, ZMAT3, BAX, DDB2, MDM2, SESN1	0.00000
U-2932	GO:0007010	Cytoskeleton organisation	TUBB2B, TUBB2A, TUBA1A, TUBB4A	0.00075
	hsa04540	Gap junction	TUBB2B, TUBB2A, TUBA1A, TUBB4A	0.00041

Top 5 GO and KEGG pathways significantly enriched in upregulated genes for each cell line after treatment with the combination of ^177^Lu-NNV003 and olaparib.

*DDB2* encodes a damage specific DNA binding protein that participates in nucleotide excision repair of DNA [40]. However, under distinct conditions, *DDB2* upregulation could increase the susceptibility of cells to detrimental genome stability [41]. *MDM2* is a proto-oncogene commonly overexpressed in tumour cells. It inhibits p53 mediated cell cycle arrest and apoptosis [42]. *SESN1*, also highly differentially expressed in GRANTA-519, encodes a protein that mediates p53-dependent inhibition of cell growth by activating AMP-activated protein kinase on detection of radiation induced DNA damage and oxidative stress causing regeneration of antioxidant proteins [43].

Genes upregulated in U-2932 cells were shown to be involved in cytoskeleton organisation and enriched in the gap junction KEGG pathway (Table 1). Genes upregulated in REC-1, SU-DHL-4 and WSU-DLCL-2 cells were not significantly enriched in any GO terms or KEGG pathway.

Downregulated genes in DOHH-2 and SU-DHL-4 cells were commonly associated with the cell division process and the cell cycle pathway, while those in WSU-DLCL-2 cells were enriched in the canonical glycolysis process and the central carbon metabolism in cancer KEGG pathway (Table 2). Genes downregulated in GRANTA-519, OCI-LY-10, REC-1 and U-2932 cells were not significantly enriched in any GO terms or KEGG pathway.

**Table 2:** Enriched pathways in downregulated genes.

Cell line	Term	Biological process	Downregulated ‘hit’ genes	p value
DOHH-2	GO:0051301	Cell division	CCNB1, FAM83D, CDCA8, CCNB2, NEK2, PSRC1, BUB1, TPX2, CDCA2, AURKA, CDC20, PTTG1, UBE2C, CDCA3	0.0000
	GO:0007067	Mitotic nuclear division	FAM83D, CCNB2, PLK1, NEK2, BUB1, TPX2, CDCA2, AURKA, CDC20, PTTG1, CDCA3	0.0000
	GO:0000086	G2/M transition of mitotic cell cycle	CCNB1, CCNB2, PLK1, NEK2, TPX2, AURKA, HMMR	0.0000
	GO:0031145	Anaphase-promoting complex-dependent catabolic process	CCNB1, PLK1, AURKA, CDC20, PTTG1, UBE2C	0.0000
	GO:0042787	Protein ubiquitination involved in ubiquitin-dependent protein catabolic process	CCNB1, PLK1, AURKA, CDC20, PTTG1, UBE2C	0.0000
	hsa04114	Oocyte meiosis	CCNB1, CCNB2, PLK1, BUB1, AURKA, CDC20, PTTG1	0.0000
	hsa04110	Cell cycle	CCNB1, CCNB2, PLK1, BUB1, CDC20, PTTG1	0.0000
	hsa04914	Progesterone-mediated oocyte maturation	CCNB1, CCNB2, PLK1, BUB1	0.0002
SU-DHL-4	GO:1904668	Positive regulation of ubiquitin protein ligase activity	PLK1, CDC20, UBE2C, UBE2S	0.0000
	GO:0051301	Cell division	CCNB1, FAM83D, PSRC1, KIF18B, CDC20, UBE2C, UBE2S, REEP4, CDCA3	0.0000
	GO:0051439	Regulation of ubiquitin-protein ligase activity involved in mitotic cell cycle	CCNB1, PLK1, CDC20, UBE2C	0.0001
	GO:0000281	Mitotic cytokinesis	KIF23, CENPA, PLK1, KIF20A	0.0001
	GO:0031145	Anaphase-promoting complex-dependent catabolic process	CCNB1, PLK1, CDC20, UBE2C, UBE2S	0.0001
WSU-DLCL-2	GO:0061621	Canonical glycolysis	PFKFB4, PFKFB3, ALDOC, HK2	0.0003
	hsa05230	Central carbon metabolism in cancer	SLC16A3, PDK1, SLC2A1, HK2, MYC	0.0008

Top 5 GO and KEGG pathways significantly enriched in downregulated genes for each cell line after treatment with the combination of ^177^Lu-NNV003 and olaparib.

Downregulated genes: *PSRC1*, *PLK1*, *KIF20A*, *CDC20*, *HILPDA* and *FAM83D*, were significantly enriched in the KEGG pathway ‘cell cycle’ and were expressed in DOHH-2, SU-DHL-4 and WSU-DLCL-2 cells. These genes are involved in mitotic cell cycle progression by mediating amongst other microtubule bundle formation [44–46].

### Mutation of genes related to DNA damage repair

Mutations in genes related to DNA damage repair might explain the difference in single agent sensitivity and the combination outcomes observed. The mRNA sequencing data was used to check for mutations in relevant genes (S4 Table) [47–49]. *TP53* was mutated in two cell lines; in REC-1, a nonsense mutation at position p.Q317* that created a stop codon (COSM1709728) and a G>A change in p.G245 that caused a glycine to aspartic acid change. In U-2932, a cysteine was changed with a tyrosine in position p.C176Y in *TP53*. In GRANTA-519, a mutation in position p.R2832C of *ATM* (COSM1351027) caused an arginine to cysteine change. *RAD51C* was mutated in DOHH-2, where the amino acid proline was replaced by a glutamine in position p.P127Q, which is expected to affect the protein function or structure. There was not found any relation between mutated genes and mRNA levels (S4 Table). S5 Table shows a summary of the mutations found in each cell-line.

## Discussion

**Fig 7:**
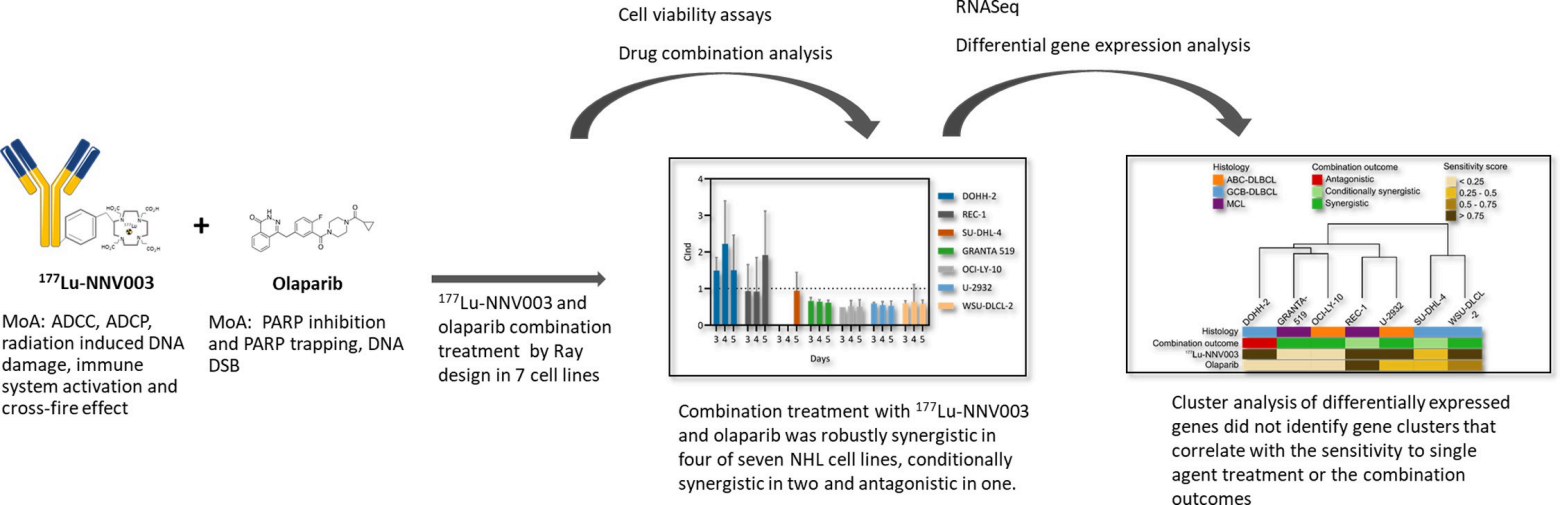
Summary of methods and results. Summary of treatments and methods used, and results obtained from the current study on the effects of combining ^177^Lu-NNV003 and olaparib in vitro. The radioimmunoconjugate ^177^Lu-NNV003 was combined with the PARP inhibitor olaparib using different concentrations of the agents in seven different cell lines (2 Mantle Cell Lymphoma and 5 DLBL cell lines). A ray design with five rays (2 rays with single agents and 3 rays with a combination of different concentrations of each agent) was selected for the study. Cell viability after treatment was measured and combination indexes for each data point measured were calculated. RNAseq and differential gene expression analysis was performed for all cell lines. Cluster analysis did not identify any specific gene sets with expression profiles that correlated with single agent treatment or combination outcome.

We have shown that the combination of the β emitting anti-CD37 RIT ^177^Lu-NNV003 and the PARP inhibitor olaparib was robustly synergistic in four of seven NHL cell lines, conditionally synergistic in two and antagonistic in one. The outcome of the combination was dependent on the ratio of the two drugs, the concentration of the mixture, and the time of measurement. Cluster analysis of differentially expressed genes did not identify gene clusters that correlate with the sensitivity to single agent treatment, CD37 expression or the combination outcomes. A summary of the methods used and results found is presented in Fig 7.

The dependence of combination outcome on the day of measurement demonstrates the importance of optimising the schedule for combination treatments. In addition, our study suggests that it is paramount to attain a suitable drug combination ratio and combination doses so as to obtain a synergistic combination outcome.

Studies have shown that olaparib can sensitise cells to radiation therapy [14, 22, 24]. Indeed, ongoing phase 1 clinical trials on the combination of olaparib and radiotherapy have different drug scheduling protocols where olaparib treatment is started some days or weeks before, or the same day as the radiotherapy treatment [26–29]. This would have to be tested in clinical settings with RIT, however, particularly because of the lower dose-rate of RIT than of radiotherapy. The aforementioned clinical trials have the same dose regimen; the radiotherapy dose is kept constant while the olaparib dose is escalated to obtain the maximum tolerated dose [26–29]. Our cell line findings indicate that the optimal combination outcome is not always at the highest drug concentrations. Hence, it might be that drug doses lower than the maximum tolerated dose should be investigated in an early clinical trial setting.

Radiation absorbed dose delivered from ^177^Lu-NNV003 to the cells in vitro is a function of self-irradiation absorbed dose from cell-bound activity in each individual cell, cross-irradiation absorbed dose from cell-bound activity to neighbouring cells and non-specific activity from the cell medium. Many variables can affect the absorbed dose of a given cell in a colony in vitro: sub-cellular and intra-cellular radiopharmaceutical distribution, cell spatial distribution, cell and nucleus size, clustering of cells, log-normal distribution of number of antigens etc. which makes cellular microdosimetry a challenge [50, 51]. In the current experimental set-up the highest absorbed dose was delivered during the 20–24 h incubation time with ^177^Lu-NNV003 where both the cells and the radioimmunoconjugate (RIC) were highly concentrated. After incubation, the cell suspensions were diluted 200 times with the aim of removing unbound ^177^Lu-NNV003. This generates a new dynamic equilibrium of bound-unbound RIC which results in a reduced amount of cell-bound RIC. In a study using Ramos cells incubated with ^177^Lu-lilotomab (the murine version of ^177^Lu-NNV003) Marcatili et al. [51] showed that the greatest contribution to the cell absorbed dose was due to cross-irradiation and non-specific irradiation even when the contribution of these two doses was limited to the incubation period of 18 h while the self-irradiation contribution included also the period after removal of the RIC from the medium and re-seeding of cells (up to 6 days). The experimental set-up used in the studies by Marcatili et al. was similar to the one described in this paper and therefore similar conclusions should apply. Although we expect slightly different radiation absorbed doses in cells measured at different timepoints, we assumed these differences to be negligible compared to the total radiation absorbed dose during the incubation period. Rough estimations of absorbed doses can be performed by assuming homogenous distribution of the RIC within the cell medium and mean energy of the β-particles, Auger- and conversion electrons of 0.1473 MeV [52]. The range of estimated absorbed doses during incubation time ranged from 0.11 mGy (lowest activity concentration used in all studies, corresponding to the lowest concentration in OCI-LY-10 cell line) to 93 Gy (highest activity concentration used in all studies, corresponding to the highest concentration in SUD-HL-4 cell line). Calculated absorbed doses after 200 times dilution of cell suspension and up to Day 3 represented around 1% of the absorbed doses during incubation, while changes in absorbed doses between Days 3, 4 and 5 represented around 0.5% (S6 Table). In addition, absorbed doses up to Day 3, 4 and 5 differed by approximately 0.2%. We therefore assume that differences in the combination outcome observed at the different timepoints are mainly related to the time-lapse of the molecular response of the cells to the radiation damage (most of which takes place during the incubation time with RIC) and how this molecular response interacts with the continued availability of olaparib in the cell suspension.

*CDKN1A*, *DDB2* and *SESN1* had the highest log2 fold change of 1.5, 1.1 and 1.1 respectively (S3 Table) in GRANTA-519 cells while *MDM2* had the highest log2 fold change of 1.0 in DOHH-2 cells. Of the three cell lines, OCI-LY-10 had the lowest fold change in these genes. *CDKN1A* encodes a cyclin-dependent kinase inhibitor which functions as a regulator of cell cycle progression, mediating the p53-dependent cell cycle G_1_ phase arrest, apoptosis and DNA repair in response to DNA damage.

Changes in gene expression in response to drug combination were different in the 7 cell lines which might explain the difference in the combination outcome.

The induction in *MDM2* expression in DOHH-2 possibly overcame the effects of the other co-upregulated genes, *CDKN1A*, *DDB2* and *SESN1*, making the cells continuously proliferate and thereby possibly explaining the antagonistic outcome of the combination in this cell line. Additionally, *ATM* was upregulated in this cell line that might have provided these cells with an alternative DDR strategy. However, ATM activation was not evaluated.

Pronounced upregulation of *CDKN1A* in the cell lines that responded synergistically to the drug combination could be as a consequence of its role as a tumour suppressor gene, increasing DNA damage induced apoptosis in these cells. Upregulation of the same gene in DOHH-2 cells can be explained by the reports on the conflicting role played by *CDKN1A*, as an oncogene, protecting cells against DNA damage-induced cell death. Either role is primarily dependent on the TP53 status of the cells but also dependent on the cytotoxic stimuli and cell type [39, 53, 54].

Downregulated genes as a consequence of combination treatment were enriched in processes that inhibit cell division and proliferation while inducing apoptosis. This could elaborate the synergism observed in SU-DHL-4 and WSU-DLCL-2 cells but is not in accordance with the antagonism observed in DOHH-2.

We did not detect an accurate correlation pattern of subtype histology, single agent sensitivity, CD37 expression or the combination outcome through unsupervised cluster analysis of filtered baseline gene expression. This could be a result of limited number of cell lines and little diversity in the tested samples. Further studies with a larger panel of cell-lines are warranted.

REC-1 has a nonsense mutation p.Q317* and a missense mutation p.G245D in *TP53*. The latter mutation is located in the highly conserved part of the protein and would probably affect the function [55]. The mutations in *TP53* might explain the low sensitivity to radiation and PARP inhibition, because of a compensating effect. U-2932 also has a mutation in *TP53*, p.C176Y, in the DNA binding domain, which could affect the protein structure and has been shown to inhibit apoptosis [56, 57]. Interestingly, these two cell lines clustered together when differential gene expression clustering analysis was performed. The p.R2832C mutation found in *ATM* in GRANTA-519 is situated in the PI-3 kinase domain which might impact ATM activity [58, 59]. In our study there was no difference in mRNA expression of *ATM* in the cell lines at baseline (p<0.01, multiple comparisons, Two Way ANOVA), however, ATM activity was not measured. There are conflicting evidence whether this mutation affects *ATM* expression and kinase activity [60, 61]. However, GRANTA-519 has been shown to have non-functional ATM [62], which might be due to this mutation. The lack of ATM functionality is in accordance with the high measured sensitivity to ^177^Lu-NNV003 and olaparib. Cells deficient of RAD51 are sensitive to PARP inhibition treatment [47] so the mutation in *RAD51C*, p.P127Q, found in DOHH-2 could explain the high sensitivity it has for olaparib treatment. These mutations could possibly explain the sensitivity of the mutated cells to the single drugs, but no clear association with the combination outcome was found.

## Conclusion

The combined effect of ^177^Lu-NNV003 and olaparib was synergistic in four NHL cell lines, partially synergistic in two and antagonistic in one. The effect of the combination was dependent on the concentration of each drug, showing the importance of optimising the parameters for further studies. Further *in vivo* studies evaluating the anti-tumour effect of the combination of RIT and PARP inhibition are warranted.

## Supporting information

S1 Table Drug concentrations and IC50s. Concentrations of olaparib and ^177^Lu-NNV003 used to treat cells for fixed-ratio ray design and mRNA sequencing study (corresponding to IC50 for each cell line).
(PDF)

S2 Table Upregulated and downregulated genes. Number of significantly up-regulated and down-regulated genes after combination treatment in each cell line. Some genes were common across the cell lines.
(PDF)

S3 Table Differentially expressed genes. Fold change of differentially expressed genes commonly annotated in combination treated cells.
(PDF)

S4 Table Genes related to DNA damage repair. List of genes related to DNA damage repair and heatmap of their corresponding mRNA level measured for each cell line.
(PDF)

S5 Table Mutations in genes related to DNA damage repair. Summary of all mutations found in genes related to DNA damage repair.
(PDF)

S6 Table Activity concentrations and absorbed doses. Activity concentration and absorbed doses during incubation (0 to 1 day), after 200 times dilution of cell suspension (1 day to 3 days) and after 1:1 dilution with Real Time Glo (RTG), (3 days to 5 days). Doses have been calculated for each interval separately.
(PDF)

S1 Fig Gating used in flow cytometry. Example of gating used for analysis of flow cytometry measurements. Cells were incubated with 10 μg/ml NNV003-AF647. In order to assess non-specific binding cells pre-incubated with 1 mg/ml NNV003 (Blocked). Autofluorescence was evaluated by measuring untreated cells (Blank). Autofluorescence was evaluated by measuring untreated cells (blanks). Sample shown in the example: GRANTA-519, incubated with NNV003-AF647.
(PDF)

S2 Fig Histograms from flow cytometry. Example of histograms obtained from flow cytometry measurements. Cells were incubated with 10 μg/ml NNV003-AF647. In order to assess non-specific binding cells pre-incubated with 1 mg/ml NNV003 (Blocked). Autofluorescence was evaluated by measuring untreated cells (Blank). REH cell line was used as negative control. Autofluorescence was evaluated by measuring untreated cells (blanks). Samples shown in the example: GRANTA-519, REH and REC-1 cells incubated with NNV003-AF647, GRANTA-519 pre-incubated with excess NNV003 and later incubated with NNV003-AF647 (Blocked) and untreated GRANTA-519 (Blank).
(PDF)

S3 Fig Pilot scheduling study. Dose response curves of Granta-519 cells treated with IC50 of ^177^Lu-NNV003 and olaparib, added 4 h before, 24 h after or at the same time as ^177^Lu-NNV003.
(PDF)

S4 Fig Filtering strategy. Flow chart of filtering methods used for gene expression analysis.
(PDF)

S5 Fig Dose response curves as function of olaparib concentration. Dose response curves of seven cell lines treated with olaparib in combination with ^177^Lu-NNV003; Ray 1, Ray 2, Ray 3 and Ray 4 as a function of olaparib concentration. Data points shown as average and error bars = SD. The experiments in DOHH-2 and WSU-DLCL-2 cells were performed twice (marked A and B).
(PDF)

S6 Fig Dose response curves as function of ^177^Lu-NNV003 concentration. Dose response curves of seven cell lines treated with olaparib in combination with ^177^Lu-NNV003; Ray 2, Ray 3, Ray 4 and Ray 5 as a function of 177Lu-NNV003 concentration. Data points shown as average and error bars = SD. The experiments in DOHH-2 and WSU-DLCL-2 cells were performed twice (marked A and B).
(PDF)

## References

[1] Swerdlow SH, Campo E, Pileri SA, Harris NL, Stein H, Siebert R, et al. The 2016 revision of the World Health Organization classification of lymphoid neoplasms. Blood. 2016;127(20):2375–90. Epub 2016/03/17. doi: 10.1182/blood-2016-01-643569 ; PubMed Central PMCID: PMC4874220. 26980727 PMC4874220

[2] Choi M, Kipps T, Kurzrock R. ATM Mutations in Cancer: Therapeutic Implications. Mol Cancer Ther. 2016;15(8):1781–91. Epub 2016/07/15. doi: 10.1158/1535-7163.MCT-15-0945 . 27413114

[3] Wang X, Huang H, Young KH. The PTEN tumor suppressor gene and its role in lymphoma pathogenesis. Aging. 2015;7(12):1032–49. Epub 2015/12/15. doi: 10.18632/aging.100855 ; PubMed Central PMCID: PMC4712330. 26655726 PMC4712330

[4] Xu-Monette ZY, Medeiros LJ, Li Y, Orlowski RZ, Andreeff M, Bueso-Ramos CE, et al. Dysfunction of the TP53 tumor suppressor gene in lymphoid malignancies. Blood. 2012;119(16):3668–83. Epub 2012/01/26. doi: 10.1182/blood-2011-11-366062 ; PubMed Central PMCID: PMC3335376. 22275381 PMC3335376

[5] Fece de la Cruz F, Gapp BV, Nijman SM. Synthetic lethal vulnerabilities of cancer. Annual review of pharmacology and toxicology. 2015;55:513–31. Epub 2014/10/24. doi: 10.1146/annurev-pharmtox-010814-124511 . 25340932

[6] Parvin S, Ramirez-Labrada A, Aumann S, Lu X, Weich N, Santiago G, et al. LMO2 Confers Synthetic Lethality to PARP Inhibition in DLBCL. Cancer Cell. 2019;36(3):237–49 e6. Epub 2019/08/27. doi: 10.1016/j.ccell.2019.07.007 ; PubMed Central PMCID: PMC6752209. 31447348 PMC6752209

[7] Bochum S, Berger S, Martens UM. Olaparib. Recent results in cancer research Fortschritte der Krebsforschung Progres dans les recherches sur le cancer. 2018;211:217–33. Epub 2018/08/03. doi: 10.1007/978-3-319-91442-8_15 . 30069770

[8] Livraghi L, Garber JE. PARP inhibitors in the management of breast cancer: current data and future prospects. BMC Med. 2015;13:188. Epub 2015/08/14. doi: 10.1186/s12916-015-0425-1 ; PubMed Central PMCID: PMC4535298. 26268938 PMC4535298

[9] Williamson CT, Muzik H, Turhan AG, Zamo A, O’Connor MJ, Bebb DG, et al. ATM Deficiency Sensitizes Mantle Cell Lymphoma Cells to Poly(ADP-Ribose) Polymerase-1 Inhibitors. Molecular Cancer Therapeutics. 2010;9(2):347–57. doi: 10.1158/1535-7163.MCT-09-0872 PubMed PMID: WOS:201244597200010. 20124459 PMC3729269

[10] Ahmed M, Li L, Pinnix C, Dabaja B, Nomie K, Lam L, et al. ATM mutation and radiosensitivity: An opportunity in the therapy of mantle cell lymphoma. Crit Rev Oncol Hematol. 2016;107:14–9. Epub 2016/11/09. doi: 10.1016/j.critrevonc.2016.08.008 . 27823642

[11] Fang NY, Greiner TC, Weisenburger DD, Chan WC, Vose JM, Smith LM, et al. Oligonucleotide microarrays demonstrate the highest frequency of ATM mutations in the mantle cell subtype of lymphoma. Proc Natl Acad Sci U S A. 2003;100(9):5372–7. Epub 2003/04/17. doi: 10.1073/pnas.0831102100 ; PubMed Central PMCID: PMC154352. 12697903 PMC154352

[12] Grønbæk K, Worm J, Ralfkiaer E, Ahrenkiel V, Hokland P, Guldberg P. ATM mutations are associated with inactivation of theARF-TP53 tumor suppressor pathway in diffuse large B-cell lymphoma. Blood. 2002;100(4):1430–7. doi: 10.1182/blood-2002-02-0382 12149228

[13] Soumerai JD, Zelenetz AD, Moskowitz CH, Palomba ML, Hamlin PA Jr., Noy A, et al. The PARP Inhibitor Veliparib Can Be Safely Added to Bendamustine and Rituximab and Has Preliminary Evidence of Activity in B-Cell Lymphoma. Clin Cancer Res. 2017;23(15):4119–26. Epub 2017/03/21. doi: 10.1158/1078-0432.CCR-16-3068 ; PubMed Central PMCID: PMC5541854. 28314788 PMC5541854

[14] Senra JM, Telfer BA, Cherry KE, McCrudden CM, Hirst DG, O’Connor MJ, et al. Inhibition of PARP-1 by Olaparib (AZD2281) Increases the Radiosensitivity of a Lung Tumor Xenograft. Molecular Cancer Therapeutics. 2011;10(10):1949–58. doi: 10.1158/1535-7163.MCT-11-0278 PubMed PMID: WOS:000295968200019. 21825006 PMC3192032

[15] van Vuurden DG, Hulleman E, Meijer OLM, Wedekind LE, Kool M, Witt H, et al. PARP inhibition sensitizes childhood high grade glioma, medulloblastoma and ependymoma to radiation. Oncotarget. 2011;2(12):984–96. PubMed PMID: WOS:000299424200012. doi: 10.18632/oncotarget.362 22184287 PMC3282104

[16] Chow JPH, Man WY, Mao M, Chen H, Cheung F, Nicholls J, et al. PARP1 Is Overexpressed in Nasopharyngeal Carcinoma and Its Inhibition Enhances Radiotherapy. Molecular Cancer Therapeutics. 2013;12(11):2517–28. doi: 10.1158/1535-7163.MCT-13-0010 PubMed PMID: WOS:000326886000021. 23979918

[17] Gani C, Coackley C, Kumareswaran R, Schutze C, Krause M, Zafarana G, et al. In vivo studies of the PARP inhibitor, AZD-2281, in combination with fractionated radiotherapy: An exploration of the therapeutic ratio. Radiother Oncol. 2015;116(3):486–94. Epub 2015/08/19. doi: 10.1016/j.radonc.2015.08.003 . 26277432

[18] Verhagen CVM, de Haan R, Hageman F, Oostendorp TPD, Carli ALE, O’Connor MJ, et al. Extent of radiosensitization by the PARP inhibitor olaparib depends on its dose, the radiation dose and the integrity of the homologous recombination pathway of tumor cells. Radiotherapy and Oncology. 2015;116(3):358–65. doi: 10.1016/j.radonc.2015.03.028 PubMed PMID: WOS:259811329700005. 25981132

[19] Mangoni M, Sottili M, Salvatore G, Meattini I, Desideri I, Greto D, et al. Enhancement of Soft Tissue Sarcoma Cell Radiosensitivity by Poly(ADP-ribose) Polymerase-1 Inhibitors. Radiation Research. 2018;190(5):464–72. doi: 10.1667/RR15035.1 PubMed PMID: WOS:000452084900002. 30067444

[20] Mao YZ, Huang X, Shuang ZY, Lin GH, Wang J, Duan FT, et al. PARP inhibitor olaparib sensitizes cholangiocarcinoma cells to radiation. Cancer Medicine. 2018;7(4):1285–96. doi: 10.1002/cam4.1318 PubMed PMID: WOS:000430663300034. 29479816 PMC5911590

[21] Parsels LA, Karnak D, Parsels JD, Zhang Q, Velez-Padilla J, Reichert ZR, et al. PARP1 Trapping and DNA Replication Stress Enhance Radiosensitization with Combined WEE1 and PARP Inhibitors. Molecular Cancer Research. 2018;16(2):222–32. doi: 10.1158/1541-7786.MCR-17-0455 PubMed PMID: WOS:291335923200004. 29133592 PMC5805596

[22] Nile DL, Rae C, Hyndman IJ, Gaze MN, Mairs RJ. An evaluation in vitro of PARP-1 inhibitors, rucaparib and olaparib, as radiosensitisers for the treatment of neuroblastoma. Bmc Cancer. 2016;16. doi: 10.1186/s12885-016-2056-0 PubMed PMID: WOS:000384167200001. 27515310 PMC4982014

[23] Tesson M, Rae C, Nixon C, Babich JW, Mairs RJ. Preliminary evaluation of prostate-targeted radiotherapy using I-131-MIP-1095 in combination with radiosensitising chemotherapeutic drugs. Journal of Pharmacy and Pharmacology. 2016;68(7):912–21. doi: 10.1111/jphp.12558 PubMed PMID: WOS:000379942000007. 27139157 PMC5298040

[24] Schaefer NG, James E, Wahl RL. Poly(ADP-ribose) polymerase inhibitors combined with external beam and radioimmunotherapy to treat aggressive lymphoma. Nucl Med Commun. 2011;32(11):1046–51. Epub 2011/10/01. doi: 10.1097/MNM.0b013e32834a369b ; PubMed Central PMCID: PMC4337870. 21956491 PMC4337870

[25] Portwood SM, Cantella MC, Cronin TL, Wang ES. Addition of the PARP Inhibitor, Talazoparib, to Gemtuzumab Ozogamicin Significantly Enhances Anti-Leukemic Activity in Human CD33+ Acute Myeloid Leukemia. Blood. 2019;134(Supplement_1):1371-. doi: 10.1182/blood-2019-130427

[26] Karam SD, Reddy K, Blatchford PJ, Waxweiler T, DeLouize AM, Oweida A, et al. Final Report of a Phase I Trial of Olaparib with Cetuximab and Radiation for Heavy Smoker Patients with Locally Advanced Head and Neck Cancer. Clinical Cancer Research. 2018. doi: 10.1158/1078-0432.CCR-18-0467 30084837 PMC6873707

[27] Fulton B, Short SC, James A, Nowicki S, McBain C, Jefferies S, et al. PARADIGM-2: Two parallel phase I studies of olaparib and radiotherapy or olaparib and radiotherapy plus temozolomide in patients with newly diagnosed glioblastoma, with treatment stratified by MGMT status. Clin Transl Radiat Oncol. 2018;8:12–6. Epub 2018/03/30. doi: 10.1016/j.ctro.2017.11.003 ; PubMed Central PMCID: PMC5862667. 29594237 PMC5862667

[28] de Haan R, van Werkhoven E, van den Heuvel MM, Peulen HMU, Sonke GS, Elkhuizen P, et al. Study protocols of three parallel phase 1 trials combining radical radiotherapy with the PARP inhibitor olaparib. BMC Cancer. 2019;19(1):901. Epub 2019/09/11. doi: 10.1186/s12885-019-6121-3 ; PubMed Central PMCID: PMC6734274. 31500595 PMC6734274

[29] Lesueur P, Lequesne J, Grellard JM, Dugue A, Coquan E, Brachet PE, et al. Phase I/IIa study of concomitant radiotherapy with olaparib and temozolomide in unresectable or partially resectable glioblastoma: OLA-TMZ-RTE-01 trial protocol. BMC Cancer. 2019;19(1):198. Epub 2019/03/06. doi: 10.1186/s12885-019-5413-y ; PubMed Central PMCID: PMC6399862. 30832617 PMC6399862

[30] Maaland AF, Heyerdahl H, O’Shea A, Eiriksdottir B, Pascal V, Andersen JT, et al. Targeting B-cell malignancies with the beta-emitting anti-CD37 radioimmunoconjugate 177Lu-NNV003. European Journal of Nuclear Medicine and Molecular Imaging. 2019;46(11):2311–21. doi: 10.1007/s00259-019-04417-1 31309259 PMC6717602

[31] Lindmo T, Boven E, Cuttitta F, Fedorko J, Bunn PA Jr. Determination of the immunoreactive fraction of radiolabeled monoclonal antibodies by linear extrapolation to binding at infinite antigen excess. J Immunol Methods. 1984;72(1):77–89. doi: 10.1016/0022-1759(84)90435-6 6086763

[32] Tomska K, Kurilov R, Lee KS, Hullein J, Lukas M, Sellner L, et al. Drug-based perturbation screen uncovers synergistic drug combinations in Burkitt lymphoma. Sci Rep. 2018;8(1):12046. Epub 2018/08/15. doi: 10.1038/s41598-018-30509-3 ; PubMed Central PMCID: PMC6089937. 30104685 PMC6089937

[33] Mateo J, Moreno V, Gupta A, Kaye SB, Dean E, Middleton MR, et al. An Adaptive Study to Determine the Optimal Dose of the Tablet Formulation of the PARP Inhibitor Olaparib. Target Oncol. 2016;11(3):401–15. Epub 2016/05/14. doi: 10.1007/s11523-016-0435-8 . 27169564

[34] Straetemans R, O’Brien T, Wouters L, Van Dun J, Janicot M, Bijnens L, et al. Design and Analysis of Drug Combination Experiments. Biometrical Journal. 2005;47(3):299–308. doi: 10.1002/bimj.200410124 16053254

[35] Foucquier J, Guedj M. Analysis of drug combinations: current methodological landscape. Pharmacol Res Perspect. 2015;3(3):e00149. Epub 2015/07/15. doi: 10.1002/prp2.149 ; PubMed Central PMCID: PMC4492765. 26171228 PMC4492765

[36] Grabovsky Y, Tallarida RJ. Isobolographic analysis for combinations of a full and partial agonist: curved isoboles. J Pharmacol Exp Ther. 2004;310(3):981–6. Epub 2004/06/04. doi: 10.1124/jpet.104.067264 . 15175417

[37] Huang DW, Sherman BT, Tan Q, Collins JR, Alvord WG, Roayaei J, et al. The DAVID Gene Functional Classification Tool: a novel biological module-centric algorithm to functionally analyze large gene lists. Genome biology. 2007;8(9):R183. Epub 2007/09/06. doi: 10.1186/gb-2007-8-9-r183 ; PubMed Central PMCID: PMC2375021. 17784955 PMC2375021

[38] Rodland GE, Melhus K, Generalov R, Gilani S, Bertoni F, Dahle J, et al. The Dual Cell Cycle Kinase Inhibitor JNJ-7706621 Reverses Resistance to CD37-Targeted Radioimmunotherapy in Activated B Cell Like Diffuse Large B Cell Lymphoma Cell Lines. Front Oncol. 2019;9:1301. Epub 2019/12/19. doi: 10.3389/fonc.2019.01301 ; PubMed Central PMCID: PMC6897291. 31850205 PMC6897291

[39] Al Bitar S, Gali-Muhtasib H. The Role of the Cyclin Dependent Kinase Inhibitor p21(cip1/waf1) in Targeting Cancer: Molecular Mechanisms and Novel Therapeutics. Cancers. 2019;11(10). Epub 2019/10/03. doi: 10.3390/cancers11101475 ; PubMed Central PMCID: PMC6826572. 31575057 PMC6826572

[40] Stoyanova T, Roy N, Kopanja D, Bagchi S, Raychaudhuri P. DDB2 decides cell fate following DNA damage. Proceedings of the National Academy of Sciences of the United States of America. 2009;106(26):10690–5. Epub 2009/06/23. doi: 10.1073/pnas.0812254106 ; PubMed Central PMCID: PMC2705559. 19541625 PMC2705559

[41] Puumalainen MR, Lessel D, Ruthemann P, Kaczmarek N, Bachmann K, Ramadan K, et al. Chromatin retention of DNA damage sensors DDB2 and XPC through loss of p97 segregase causes genotoxicity. Nature communications. 2014;5:3695. Epub 2014/04/29. doi: 10.1038/ncomms4695 ; PubMed Central PMCID: PMC4007632. 24770583 PMC4007632

[42] Giono LE, Resnick-Silverman L, Carvajal LA, St Clair S, Manfredi JJ. Mdm2 promotes Cdc25C protein degradation and delays cell cycle progression through the G2/M phase. Oncogene. 2017;36(49):6762–73. Epub 2017/08/15. doi: 10.1038/onc.2017.254 ; PubMed Central PMCID: PMC6002854. 28806397 PMC6002854

[43] Budanov AV, Lee JH, Karin M. Stressin’ Sestrins take an aging fight. EMBO Mol Med. 2010;2(10):388–400. Epub 2010/09/30. doi: 10.1002/emmm.201000097 ; PubMed Central PMCID: PMC3166214. 20878915 PMC3166214

[44] Hsieh WJ, Hsieh SC, Chen CC, Wang FF. Human DDA3 is an oncoprotein down-regulated by p53 and DNA damage. Biochemical and biophysical research communications. 2008;369(2):567–72. Epub 2008/02/23. doi: 10.1016/j.bbrc.2008.02.047 . 18291097

[45] Kumar S, Sharma AR, Sharma G, Chakraborty C, Kim J. PLK-1: Angel or devil for cell cycle progression. Biochimica et biophysica acta. 2016;1865(2):190–203. Epub 2016/02/24. doi: 10.1016/j.bbcan.2016.02.003 . 26899266

[46] Wu WD, Yu KW, Zhong N, Xiao Y, She ZY. Roles and mechanisms of Kinesin-6 KIF20A in spindle organization during cell division. European journal of cell biology. 2019;98(2–4):74–80. Epub 2018/12/24. doi: 10.1016/j.ejcb.2018.12.002 . 30579662

[47] McCabe N, Turner NC, Lord CJ, Kluzek K, Bialkowska A, Swift S, et al. Deficiency in the repair of DNA damage by homologous recombination and sensitivity to poly(ADP-ribose) polymerase inhibition. Cancer Res. 2006;66(16):8109–15. Epub 2006/08/17. doi: 10.1158/0008-5472.CAN-06-0140 . 16912188

[48] Lord CJ, Ashworth A. BRCAness revisited. Nat Rev Cancer. 2016;16(2):110–20. Epub 2016/01/19. doi: 10.1038/nrc.2015.21 . 26775620

[49] Zimmermann M, Murina O, Reijns MAM, Agathanggelou A, Challis R, Tarnauskaite Z, et al. CRISPR screens identify genomic ribonucleotides as a source of PARP-trapping lesions. Nature. 2018;559(7713):285–9. Epub 2018/07/06. doi: 10.1038/s41586-018-0291-z ; PubMed Central PMCID: PMC6071917. 29973717 PMC6071917

[50] Dahle J, Krogh C, Melhus KB, Borrebaek J, Larsen RH, Kvinnsland Y. In vitro cytotoxicity of low-dose-rate radioimmunotherapy by the alpha-emitting radioimmunoconjugate Thorium-227-DOTA-rituximab. Int J Radiat Oncol Biol Phys. 2009;75(3):886–95. doi: S0360-3016(09)00661-0 [pii]; doi: 10.1016/j.ijrobp.2009.04.062 19679402

[51] Marcatili S, Pichard A, Courteau A, Ladjohounlou R, Navarro-Teulon I, Repetto-Llamazares A, et al. Realistic multi-cellular dosimetry for (177)Lu-labelled antibodies: model and application. Phys Med Biol. 2016;61(19):6935–52. Epub 2016/09/13. doi: 10.1088/0031-9155/61/19/6935 . 27617585

[52] Center NND. National Nuclear Data Center, ENSDF Decay Data in the MIRD (Medical Internal Radiation Dose) Format for 177Lu 2012 [updated 10/16/2012; cited 2012 10/16/2012]. Available from: http://www.nndc.bnl.gov/useroutput/177lu_mird.html.

[53] Wang Y, Blandino G, Givol D. Induced p21waf expression in H1299 cell line promotes cell senescence and protects against cytotoxic effect of radiation and doxorubicin. Oncogene. 1999;18(16):2643–9. Epub 1999/06/03. doi: 10.1038/sj.onc.1202632 . 10353608

[54] Barboza JA, Liu G, Ju Z, El-Naggar AK, Lozano G. p21 delays tumor onset by preservation of chromosomal stability. Proceedings of the National Academy of Sciences of the United States of America. 2006;103(52):19842–7. Epub 2006/12/16. doi: 10.1073/pnas.0606343104 ; PubMed Central PMCID: PMC1702317. 17170138 PMC1702317

[55] Srivastava S, Zou Z, Pirollo K, Blattner W, Chang EH. Germ-line transmission of a mutated p53 gene in a cancer-prone family with Li–Fraumeni syndrome. Nature. 1990;348(6303):747–9. doi: 10.1038/348747a0 2259385

[56] Bergamaschi D, Gasco M, Hiller L, Sullivan A, Syed N, Trigiante G, et al. p53 polymorphism influences response in cancer chemotherapy via modulation of p73-dependent apoptosis. Cancer Cell. 2003;3(4):387–402. doi: 10.1016/s1535-6108(03)00079-5 12726864

[57] Mullany LK, Wong KK, Marciano DC, Katsonis P, King-Crane ER, Ren YA, et al. Specific TP53 Mutants Overrepresented in Ovarian Cancer Impact CNV, TP53 Activity, Responses to Nutlin-3a, and Cell Survival. Neoplasia. 2015;17(10):789–803. Epub 2015/11/21. doi: 10.1016/j.neo.2015.10.003 ; PubMed Central PMCID: PMC4656807. 26585234 PMC4656807

[58] Vořechovský I, Luo L, Dyer MJS, Catovsky D, Amlot PL, Yaxley JC, et al. Clustering of missense mutations in the ataxia-telanglectasia gene in a sporadic T-cell leukaemia. Nature Genetics. 1997;17(1):96–9. doi: 10.1038/ng0997-96 9288106

[59] Becker-Catania SG, Chen G, Hwang MJ, Wang Z, Sun X, Sanal O, et al. Ataxia-telangiectasia: phenotype/genotype studies of ATM protein expression, mutations, and radiosensitivity. Mol Genet Metab. 2000;70(2):122–33. Epub 2000/06/30. doi: 10.1006/mgme.2000.2998 . 10873394

[60] Barone G, Groom A, Reiman A, Srinivasan V, Byrd PJ, Taylor AM. Modeling ATM mutant proteins from missense changes confirms retained kinase activity. Hum Mutat. 2009;30(8):1222–30. Epub 2009/05/12. doi: 10.1002/humu.21034 . 19431188

[61] Mitui M, Nahas SA, Du LT, Yang Z, Lai CH, Nakamura K, et al. Functional and computational assessment of missense variants in the ataxia-telangiectasia mutated (ATM) gene: mutations with increased cancer risk. Hum Mutat. 2009;30(1):12–21. Epub 2008/07/18. doi: 10.1002/humu.20805 ; PubMed Central PMCID: PMC2776735. 18634022 PMC2776735

[62] Williamson CT, Kubota E, Hamill JD, Klimowicz A, Ye R, Muzik H, et al. Enhanced cytotoxicity of PARP inhibition in mantle cell lymphoma harbouring mutations in both ATM and p53. EMBO Mol Med. 2012;4(6):515–27. Epub 2012/03/15. doi: 10.1002/emmm.201200229 ; PubMed Central PMCID: PMC3443945. 22416035 PMC3443945

