# Supplementary material for: Anti-CD37 radioimmunotherapy with 177Lu-NNV003 synergizes with the PARP inhibitor olaparib in treatment of non-Hodgkin’s lymphoma in vitro
Source: PLoS One. 2022 Apr 29;17(4):e0267543. doi: 10.1371/journal.pone.0267543 (PMC9053826; doi:10.1371/journal.pone.0267543)
Supplement: S1 Table — Concentrations of olaparib and 177Lu-NNV003 used to treat cells for fixed-ratio ray design and mRNA sequencing study (corresponding to IC50 for each cell line). (PDF) [file pone.0267543.s001.pdf]

# Anti-CD37 radioimmunotherapy with $^{177}$Lu-NNV003 synergises with the PARP inhibitor olaparib in treatment of non-Hodgkin's lymphoma in vitro

# Supplementary

**S1 Table. Drug concentrations and IC50s.** Concentrations of olaparib and $^{177}$Lu-NNV003 used to treat cells for fixed-ratio ray design and mRNA sequencing study (corresponding to IC50 for each cell line).

| | **Olaparib** | | | **$^{177}$Lu-NNV003** | | | | | |
|---|---|---|---|---|---|---|---|---|---|
| **Cell Line** | **IC50** | **Min** | **Max** | **IC50** | | **Min** | | **Max** | |
| | (uM) | (uM) | (uM) | (ng/ml) | (MBq/ml) | (ng/ml) | (MBq/ml) | (ng/ml) | (MBq/ml) |
| **DOHH-2** | 1.31 | 0.0080 | 196.43 | 138 | 0.08 | 1.08 | 0.00059 | 20746.52 | 11.41 |
| **GRANTA-519** | 0.86 | 0.0100 | 25.81 | 189 | 0.10 | 2.4 | 0.00132 | 5667.68 | 3.12 |
| **OCI-LY-10** | 0.89 | 0.0020 | 44.48 | 41 | 0.02 | 0.1 | 0.00006 | 20206.87 | 11.11 |
| **REC-1** | 12.66 | 0.0284 | 316.58 | 287 | 0.16 | 0.66 | 0.00036 | 25800.29 | 14.19 |
| **SU-DHL-4** | 2.36 | 0.0015 | 59.01 | 177 | 0.10 | 0.11 | 0.00006 | 87092.65 | 47.90 |
| **U-2932** | 1.31 | 0.0020 | 25.81 | 111 | 0.06 | 2.4 | 0.00132 | 5667.00 | 3.12 |
| **WSU-DLCL-2** | 5.66 | 0.0360 | 31.14 | 234 | 0.13 | 1.43 | 0.00079 | 1287.03 | 0.71 |
