# Supplementary material for: Anti-CD37 radioimmunotherapy with 177Lu-NNV003 synergizes with the PARP inhibitor olaparib in treatment of non-Hodgkin’s lymphoma in vitro
Source: PLoS One. 2022 Apr 29;17(4):e0267543. doi: 10.1371/journal.pone.0267543 (PMC9053826; doi:10.1371/journal.pone.0267543)
Supplement: S2 Table — Number of significantly up-regulated and down-regulated genes after combination treatment in each cell line. Some genes were common across the cell lines. (PDF) [file pone.0267543.s002.pdf]

Anti-CD37 radioimmunotherapy with ^{177}Lu-NNV003 synergises with the PARP inhibitor olaparib in treatment of non-Hodgkin's lymphoma in vitro

Supplementary

S2 Table. Upregulated and downregulated genes. Number of significantly upregulated and downregulated genes after combination treatment in each cell line. Some genes were common across the cell lines.

Cell line	Number of genes	
	Upregulated	Downregulated
**DOHH-2**	68	29
**GRANTA-519**	59	22
**OCI-LY-10**	20	2
**REC-1**	11	21
**SU-DHL-4**	28	73
**U-2932**	22	7
**WSU-DLCL-2**	18	109
**Total**	188	209
