# Supplementary material for: Anti-CD37 radioimmunotherapy with 177Lu-NNV003 synergizes with the PARP inhibitor olaparib in treatment of non-Hodgkin’s lymphoma in vitro
Source: PLoS One. 2022 Apr 29;17(4):e0267543. doi: 10.1371/journal.pone.0267543 (PMC9053826; doi:10.1371/journal.pone.0267543)
Supplement: S3 Table — Fold change of differentially expressed genes commonly annotated in combination treated cells. (PDF) [file pone.0267543.s003.pdf]

Anti-CD37 radioimmunotherapy with ^{177}Lu-NNV003 synergises with the PARP inhibitor olaparib in treatment of non-Hodgkin's lymphoma in vitro

Supplementary

S3 Table. Differentially expressed genes. Fold change of differentially expressed genes commonly annotated in combination treated cells.

**Hit genes**	**Log2 fold-change**				
	DOHH-2	GRANTA-519	OCI-LY-10	SU-DHL-4	WSU-DLCL-2
CDKN1A	1.0	1.5	0.8	--	--
DDB2	0.7	1.1	0.8	--	--
SESN1	0.8	1.1	0.6	--	--
MDM2	1.0	0.8	0.7	--	--
PSRC1	-1.1	--	--	-0.7	-0.6
PLK1	-0.7	--	--	-0.7	-0.5
KIF20A	-1.0	--	--	-0.9	-0.8
CDC20	-0.8	--	--	-0.6	-0.6
HILPDA	-0.6	--	--	-0.6	-0.8
FAM83D	-0.8	--	--	-0.7	-0.6
