# Supplementary material for: Anti-CD37 radioimmunotherapy with 177Lu-NNV003 synergizes with the PARP inhibitor olaparib in treatment of non-Hodgkin’s lymphoma in vitro
Source: PLoS One. 2022 Apr 29;17(4):e0267543. doi: 10.1371/journal.pone.0267543 (PMC9053826; doi:10.1371/journal.pone.0267543)
Supplement: S4 Table — List of genes related to DNA damage repair and heatmap of their corresponding mRNA level measured for each cell line. (PDF) [file pone.0267543.s004.pdf]

Anti-CD37 radioimmunotherapy with ^{177}Lu-NNV003 synergises with the PARP inhibitor olaparib in treatment of non-Hodgkin's lymphoma in vitro

Supplementary

S4 Table. Genes related to DNA damage repair. List of genes related to DNA damage repair and heatmap of their corresponding mRNA level measured for each cell line.

Gene	DOHH-2	GRANTA-519	OCI-LY-10	REC-1	SU-DHL-4	U-2932	WSU-DLCL-4
ATM	8.70	17.26	24.28	16.91	13.62	18.86	20.63
ATR	4.92	8.52	3.01	5.17	6.57	18.23	3.93
CHK1	30.80	30.51	19.06	48.91	21.24	16.02	27.87
CHK2	28.27	21.43	12.55	18.95	9.81	21.02	18.49
DSS1	416.58	143.81	247.79	438.16	310.58	269.16	220.16
FANCA	17.83	21.25	12.77	10.46	14.67	41.21	33.21
FANCC	3.52	6.86	6.70	5.87	2.04	6.58	1.54
NBS1	46.78	37.41	29.54	27.79	10.20	25.16	15.56
RAD51	20.95	14.65	11.53	18.56	13.33	20.56	21.11
RAD54	22.34	16.78	15.45	25.95	22.08	14.23	13.24
RPA1	48.74	29.98	49.34	33.92	26.43	103.05	54.08
BRCA2	2.65	10.12	6.14	1.26	1.05	2.31	1.44
DDB1	76.53	86.24	87.06	58.62	40.80	120.35	52.90
XAB2	42.06	36.57	37.94	66.94	55.28	42.03	33.13
XRCC1	25.07	27.66	38.54	17.53	18.96	19.46	36.86
BARD1	10.49	12.76	7.68	4.12	8.39	16.00	8.64
BRCA1	14.87	17.87	8.33	9.67	3.32	20.04	10.65
EMSY	6.70	6.08	5.82	6.78	5.90	5.37	8.75
PALB2	8.09	9.60	7.08	3.91	1.28	8.30	4.65
PSMC3IP	24.36	19.03	7.58	10.34	12.64	13.33	16.65
RAD51B	7.79	8.28	6.25	4.05	7.39	5.73	12.93
RAD51C	30.31	40.80	30.61	43.49	17.09	43.88	18.72
RAD51D	13.41	14.07	3.63	10.06	7.85	5.86	5.36
RBBP8	37.39	15.49	22.84	29.17	24.13	59.28	12.93
TONSL	15.81	17.17	10.75	19.47	18.13	8.96	12.34
XRCC2	7.88	11.55	2.72	6.58	1.47	7.65	1.28
XRCC3	22.96	34.73	5.66	19.78	10.70	25.78	13.16
COMMD1	29.90	36.96	35.34	49.22	17.93	25.41	12.83
FAAP24	4.04	4.65	3.06	3.24	2.80	3.47	4.71
FANCD2	18.48	22.50	24.17	10.27	13.85	18.27	21.75
FANCE	8.08	7.43	2.60	12.00	5.81	6.65	5.13
FANCM	2.74	4.41	3.97	3.32	2.18	2.31	2.50
UBE2T	94.03	63.85	44.70	43.70	71.55	73.97	75.51
EME1	14.90	10.75	7.29	9.19	3.90	7.25	10.73
HUS1	12.82	10.95	9.16	20.45	9.81	9.12	11.34
MUS81	22.71	29.73	11.96	45.23	17.05	19.69	20.01
TP53	73.38	123.10	51.83	14.78	26.42	85.25	36.80
