# Supplementary material for: Anti-CD37 radioimmunotherapy with 177Lu-NNV003 synergizes with the PARP inhibitor olaparib in treatment of non-Hodgkin’s lymphoma in vitro
Source: PLoS One. 2022 Apr 29;17(4):e0267543. doi: 10.1371/journal.pone.0267543 (PMC9053826; doi:10.1371/journal.pone.0267543)
Supplement: S5 Table — Summary of all mutations found in genes related to DNA damage repair. (PDF) [file pone.0267543.s005.pdf]

Anti-CD37 radioimmunotherapy with ^{177}Lu-NNV003 synergises with the PARP inhibitor olaparib in treatment of non-Hodgkin's lymphoma in vitro

Supplementary

S5 Table. Mutations in genes related to DNA damage repair.

Cell line	Gene	Amino acid change	dbSNP or COSMIC identification number
DOHH-2	*RAD51C*	p.P127Q	NA
GRANTA-519	*ATM*	p.R2832C	rs587779872/COSM1351027
REC-1	*TP53*	p.Q317*	COSM1709728
	TP53	p.G245D	rs121912656
U-2932	*TP53*	p.C176Y	rs786202962
