# Supplementary material for: Anti-CD37 radioimmunotherapy with 177Lu-NNV003 synergizes with the PARP inhibitor olaparib in treatment of non-Hodgkin’s lymphoma in vitro
Source: PLoS One. 2022 Apr 29;17(4):e0267543. doi: 10.1371/journal.pone.0267543 (PMC9053826; doi:10.1371/journal.pone.0267543)
Supplement: S6 Table — Activity concentration and absorbed doses during incubation (0 to 1 day), after 200 times dilution of cell suspension (1 day to 3 days) and after 1:1 dilution with Real Time Glo (RTG), (3 days to 5 days). Doses have been calculated for each interval separately. (PDF) [file pone.0267543.s006.pdf]

Anti-CD37 radioimmunotherapy with ^{177}Lu-NNV003 synergises with the PARP inhibitor olaparib in treatment of non-Hodgkin's lymphoma in vitro

Supplementary

S6 Table. Activity concentrations and absorbed doses. Activity concentration and absorbed doses during incubation (0 to 1 day), after 200 times dilution of cell suspension (1 day to 3 days) and after 1:1 dilution with Real Time Glo (RTG), (3 days to 5 days). Doses have been calculated for each interval separately.

	Activity concentration			Absorbed dose			
	At incubation	After 200x dilution	After RTG	During Incubation	ΔDay 3	ΔDay 4	ΔDay 5
	0 to 1d	1d to 3d	3d to 5d	0 to 1d	1d to 3d	3d to 4d	4d to 5d
**Cell Line**	**MBq/ml**	**MBq/ml**	**MBq/ml**	**Gy**	**Gy**	**Gy**	**Gy**
**OCI-LY-10**	5.50E-05	2.75E-07	1.38E-07	1.07E-04	9.13E-07	3.9E-07	3.51E-07
**SU-DHL-4**	47.90	0.24	0.12	92.76	0.79	0.17	0.15
