# Supplementary material for: Anti-CD37 radioimmunotherapy with 177Lu-NNV003 synergizes with the PARP inhibitor olaparib in treatment of non-Hodgkin’s lymphoma in vitro
Source: PLoS One. 2022 Apr 29;17(4):e0267543. doi: 10.1371/journal.pone.0267543 (PMC9053826; doi:10.1371/journal.pone.0267543)
Supplement: S1 Fig — Example of gating used for analysis of flow cytometry measurements. Cells were incubated with 10 μg/ml NNV003-AF647. In order to assess non-specific binding cells pre-incubated with 1 mg/ml NNV003 (Blocked). Autofluorescence was evaluated by measuring untreated cells (Blank). Autofluorescence was evaluated by measuring untreated cells (blanks). Sample shown in the example: GRANTA-519, incubated with NNV003-AF647. (PDF) [file pone.0267543.s007.pdf]

Anti-CD37 radioimmunotherapy with ^{177}Lu-NNV003 synergises with the PARP inhibitor olaparib in treatment of non-Hodgkin's lymphoma in vitro

Supplementary

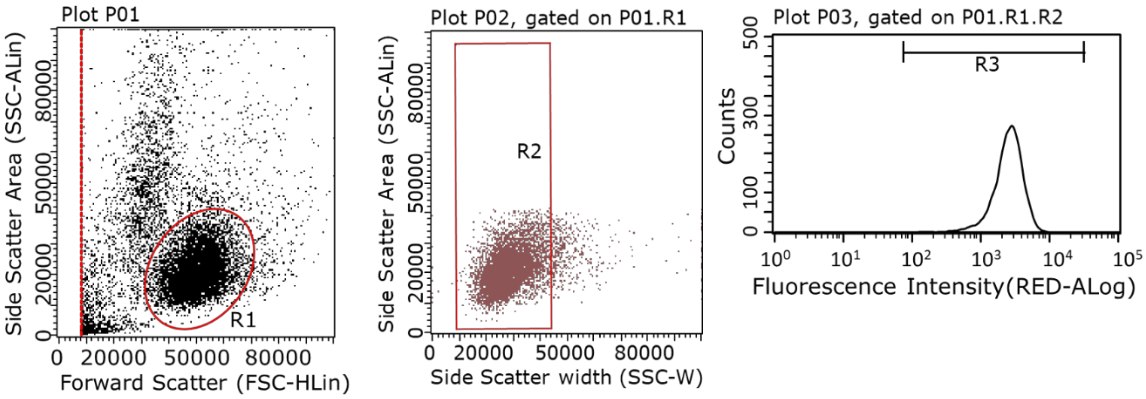

S1 Figure. Gating used in flow cytometry. Example of gating used for analysis of flow cytometry measurements. Cells were incubated with 10 µg/ml NNV003-AF647. In order to assess non-specific binding cells pre-incubated with 1 mg/ml NNV003 (Blocked). Autofluorescence was evaluated by measuring untreated cells (Blank). Autofluorescence was evaluated by measuring untreated cells (blanks). Sample shown in the example: GRANTA-519, incubated with NNV003-AF647.
