# Supplementary material for: Anti-CD37 radioimmunotherapy with 177Lu-NNV003 synergizes with the PARP inhibitor olaparib in treatment of non-Hodgkin’s lymphoma in vitro
Source: PLoS One. 2022 Apr 29;17(4):e0267543. doi: 10.1371/journal.pone.0267543 (PMC9053826; doi:10.1371/journal.pone.0267543)
Supplement: S2 Fig — Example of histograms obtained from flow cytometry measurements. Cells were incubated with 10 μg/ml NNV003-AF647. In order to assess non-specific binding cells pre-incubated with 1 mg/ml NNV003 (Blocked). Autofluorescence was evaluated by measuring untreated cells (Blank). REH cell line was used as negative control. Autofluorescence was evaluated by measuring untreated cells (blanks). Samples shown in the example: GRANTA-519, REH and REC-1 cells incubated with NNV003-AF647, GRANTA-519 pre-incubated with excess NNV003 and later incubated with NNV003-AF647 (Blocked) and untreated GRANTA-519 (Blank). (PDF) [file pone.0267543.s008.pdf]

Anti-CD37 radioimmunotherapy with ^{177}Lu-NNV003 synergises with the PARP inhibitor olaparib in treatment of non-Hodgkin's lymphoma in vitro

Supplementary

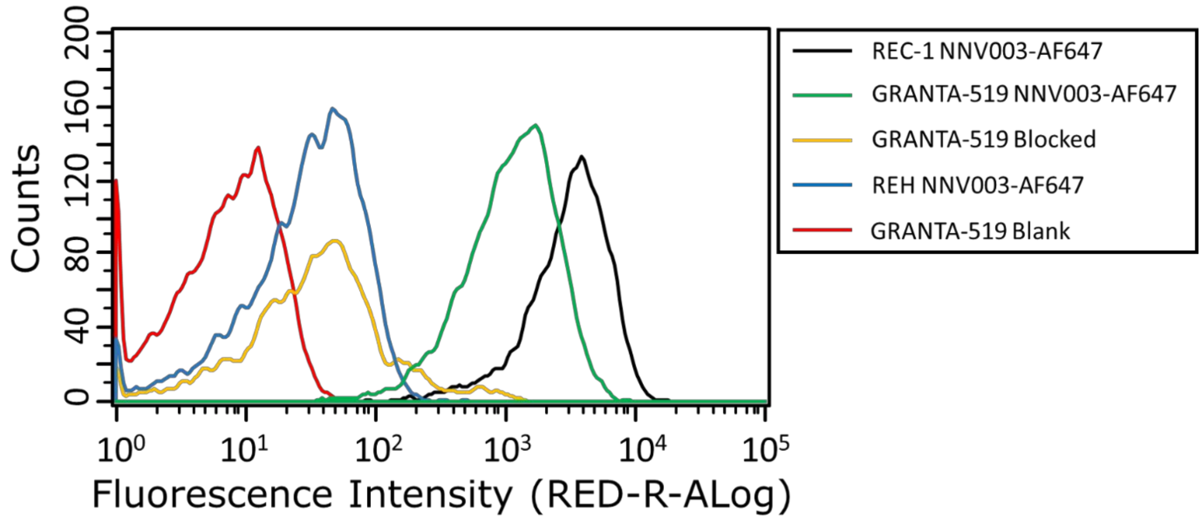

S2 Figure. Histograms from flow cytometry. Example of histograms obtained from flow cytometry measurements. Cells were incubated with 10 µg/ml NNV003-AF647. In order to assess non-specific binding cells pre-incubated with 1 mg/ml NNV003 (Blocked). Autofluorescence was evaluated by measuring untreated cells (Blank). REH cell line was used as negative control. Autofluorescence was evaluated by measuring untreated cells (blanks). Samples shown in the example: GRANTA-519, REH and REC-1 cells incubated with NNV003-AF647, GRANTA-519 pre-incubated with excess NNV003 and later incubated with NNV003-AF647 (Blocked) and untreated GRANTA-519 (Blank).
