# Supplementary material for: Anti-CD37 radioimmunotherapy with 177Lu-NNV003 synergizes with the PARP inhibitor olaparib in treatment of non-Hodgkin’s lymphoma in vitro
Source: PLoS One. 2022 Apr 29;17(4):e0267543. doi: 10.1371/journal.pone.0267543 (PMC9053826; doi:10.1371/journal.pone.0267543)
Supplement: S3 Fig — Dose response curves of Granta-519 cells treated with IC50 of 177Lu-NNV003 and olaparib, added 4 h before, 24 h after or at the same time as 177Lu-NNV003. (PDF) [file pone.0267543.s009.pdf]

Anti-CD37 radioimmunotherapy with ^{177}Lu-NNV003 synergises with the PARP inhibitor olaparib in treatment of non-Hodgkin's lymphoma in vitro

Supplementary

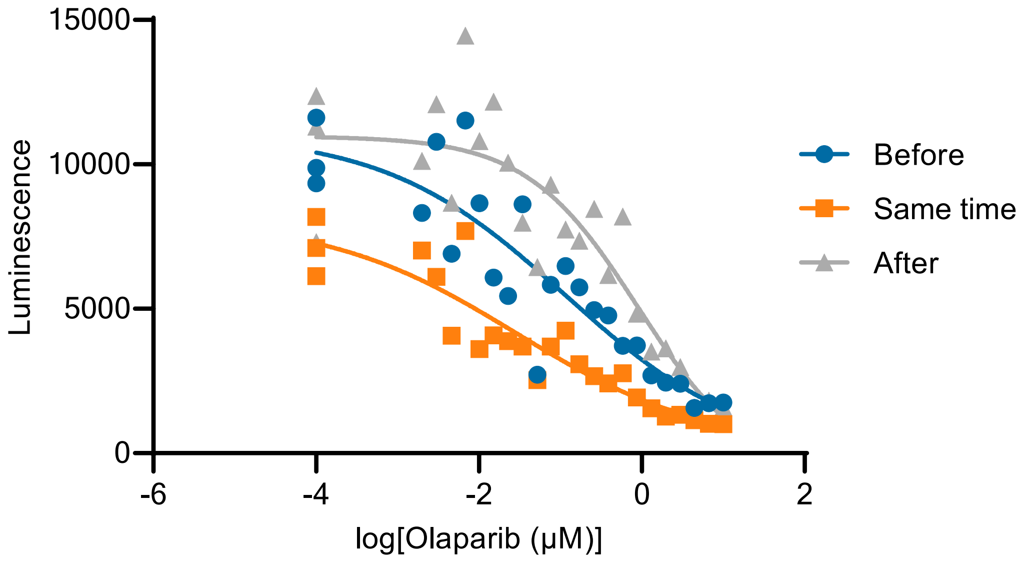

S3 Figure. Pilot scheduling study. Dose response curves of Granta-519 cells treated with IC50 of ^{177}Lu-NNV003 and olaparib, added 4 h before, 24 h after or at the same time as ^{177}Lu-NNV003.
