# Supplementary material for: Anti-CD37 radioimmunotherapy with 177Lu-NNV003 synergizes with the PARP inhibitor olaparib in treatment of non-Hodgkin’s lymphoma in vitro
Source: PLoS One. 2022 Apr 29;17(4):e0267543. doi: 10.1371/journal.pone.0267543 (PMC9053826; doi:10.1371/journal.pone.0267543)
Supplement: S6 Fig — Dose response curves of seven cell lines treated with olaparib in combination with 177Lu-NNV003; Ray 2, Ray 3, Ray 4 and Ray 5 as a function of 177Lu-NNV003 concentration. Data points shown as average and error bars = SD. The experiments in DOHH-2 and WSU-DLCL-2 cells were performed twice (marked A and B). (PDF) [file pone.0267543.s012.pdf]

Anti-CD37 radioimmunotherapy with ^{177}Lu-NNV003 synergises with the PARP inhibitor olaparib in treatment of non-Hodgkin's lymphoma in vitro

Supplementary

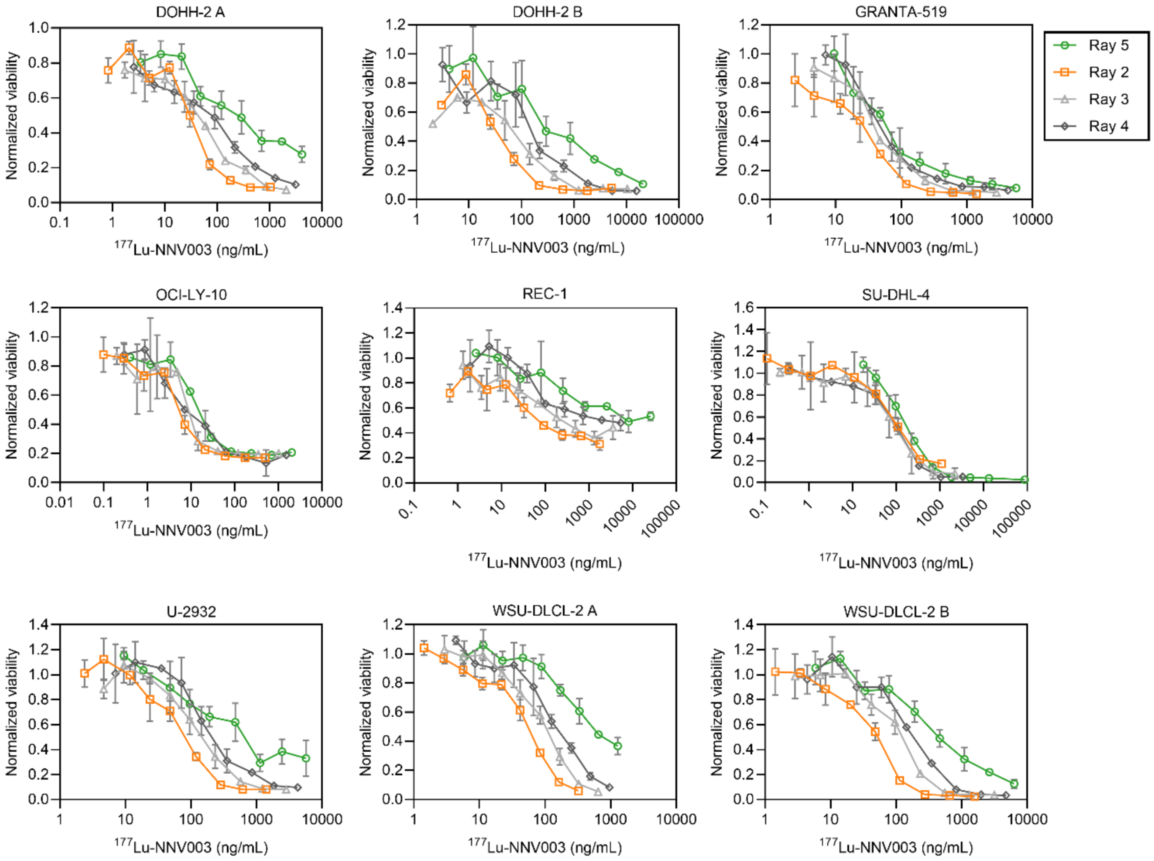

S6 Figure. Dose response curves as function of ^{177}Lu-NNV003 concentration. Dose response curves of seven cell lines treated with olaparib in combination with ^{177}Lu-NNV003; Ray 2, Ray 3, Ray 4 and Ray 5 as a function of 177Lu-NNV003 concentration. Data points shown as average and error bars= SD. The experiments in DOHH-2 and WSU-DLCL-2 cells were performed twice (marked A and B).
